# Pathways linking household food insecurity to undernutrition among under 5 children in migrant populations along the Thailand-Myanmar border: A Structural Equation Modeling approach

**DOI:** 10.1371/journal.pgph.0006516

**Published:** 2026-08-20

**Authors:** Saw Min Than, Thunwadee Suksaroj, Piyapong Janmaimool, Cheerawit Rattanapan

**Affiliations:** 1 ASEAN Institute for Health Development, Mahidol University, Salaya, Nakhon Pathom, Thailand; 2 Center for Global Health - Mahidol University, Mahidol University, Phuttamonthon, Salaya, Nakhon Pathom, Thailand; PLOS: Public Library of Science, UNITED STATES OF AMERICA

## Abstract

Child malnutrition remains a global crisis, exacerbated in border regions by Myanmar’s post-2021 conflict. This study utilized Structural Equation Modeling (SEM) to examine the pathways linking household food insecurity (HFI) to undernutrition among migrant children along the Thailand–Myanmar border. A cross-sectional survey was conducted among 384 mothers in Tak and Mae Hong Son provinces with children aged 6–59 months. Data were collected via multistage sampling, face-to-face interviews, and anthropometric assessments. HFI was measured using the HFIAS scale. Undernutrition affected 37.0% of children, with specific prevalence for stunting (18.0%), wasting (19.8%), and underweight (18.5%). HFI was prevalent in 63.8% of households and served as a central structural mediator; notably, nearly 40% of stunted children resided in severely food-insecure households. SEM results indicated that higher socio-economic status (SES) consistently improved HAZ (β = 0.230, p < .001) and WAZ (β = 0.294, p < .001). Improved water availability, safe sanitation, and higher SES were strong predictors of better HFI status across all models (β ranging from 0.127 to 0.318, p < .023). Conversely, a higher number of children under five in the household negatively impacted food security (β ranging from -0.155 to -0.159, p < .003). This improved HFI status directly enhanced dietary diversity (β = 0.183, p = .001) and HAZ (β = 0.113, p = .033). Regarding acute outcomes, fever (β = -0.147, p = .008) and diarrhea (β = -0.131, p = .049) significantly reduced WHZ, while respiratory infections (β = -0.144, p = .014) negatively influenced WAZ. Addressing undernutrition in this migrant population requires integrated multi-sectoral interventions. Strategies must prioritize economic stability and HFI mitigation to address chronic growth failure, alongside WASH infrastructure and rapid illness response to mitigate acute and composite nutritional deficits through a synergistic, cross-sectoral approach.

## 1 Introduction

Malnutrition remains one of the most pressing global health challenges, affecting millions of children worldwide and undermining national development, economic growth, and intergenerational health [1]. It encompasses both deficiency disorders (stunting, wasting, underweight, and micronutrient deficiencies) and excess weight (overweight and obesity). Undernutrition, a subset of malnutrition is defined as insufficient dietary energy and nutrient intake to meet basic physiological needs and arises from complex interactions among biological, social, and environmental determinants, including poverty, inadequate feeding practices, limited health services, and high disease burden [1–3]. A life-course approach highlights critical windows for nutrition interventions, spanning pre-conception, gestation, lactation, childhood, and adulthood, with nutritional and environmental insults at any stage potentially affecting long-term health across generations [1,4].

Child undernutrition has enduring consequences, limiting cognitive development, learning capacity, and societal contribution, while increasing susceptibility to illness and mortality [2,5]. According to the UNICEF Conceptual Framework for Maternal and Child Nutrition, undernutrition primarily arises from poor childcare practices, unstable household food security, and deficiencies in essential services such as healthcare, clean water, and sanitation [3,6,7]. Addressing child undernutrition is therefore essential for improving human capital, accelerating economic development, and reducing poverty [5].

Globally, in 2022, nearly one-quarter (22.3%) of children under five, approximately 148.1 million, were stunted, 45 million (6.8%) were acutely malnourished (wasting), and 37 million (5.6%) were overweight. Stunting rates in Least Developed Countries exceeded the global average, although wasting prevalence remained comparable [1]. Asia bore a disproportionate share of the global malnutrition burden, accounting for 70% of all wasted children under five and over half of all stunted children. Africa also carried a substantial burden, with two in five children stunted and more than 25% wasted [8].

Despite notable declines since 1990, the WHO Southeast Asia Region continued to report the highest prevalence of wasting among all WHO regions in 2022 at 14.7% (down from 17.1%), affecting nearly one in five children, while stunting remained high at 30.1% (down from 60.5%), impacting three out of ten children [9]. Sustainable Development Goal 2 (Zero Hunger) remains distant for the global community. Food insecurity was moderate or severe for 2.33 billion people (28.9%) globally in 2023. Asia accounted for the largest portion, with 1.18 billion individuals (24.8%) experiencing moderate or severe insecurity, including 467.3 million (9.8%) in severe insecurity—substantially exceeding Africa’s 847 million [1]. Southeast Asia reported the second-highest rate of moderate or severe food insecurity among Asian subregions at 17.1%, affecting 117.7 million people.

In Myanmar, ongoing conflict and instability since February 2021 has critically endangered children’s lives, driven by economic hardship, a collapsing health system, and widespread violence [10,11]. Consequently, in 2024, more than 40 deaths per 1,000 live births were reported nationally, with vulnerability highest among conflict-affected and displaced poorer families [12]. According to the humanitarian assessments, an estimated 16.2 million people, including 4.9 million children, require humanitarian assistance. Within this protracted crisis, approximately 2.6 million children are in immediate need of life-saving humanitarian and essential nutrition services, with particular urgency centered on mitigating severe vulnerabilities across conflict-affected states and sensitive border zones [13].

Migrant and refugee communities along the Thailand–Myanmar border are particularly vulnerable, often practicing suboptimal infant feeding, contributing to high rates of stunting and underweight [14]. The Border Consortium’s 2022 Nutrition Survey reported global acute malnutrition (wasting) at 3.5%, with camp-level rates ranging from 1.3% to 4.7%, and chronic malnutrition (stunting) at 21.5%, ranging from 9.4% to 33.3% by camp [15]. Another study in refugee camps reported wasting and stunting rates of 2.1% and 28.8%, respectively [16]. These findings underscore a serious public health emergency and highlight the critical need for targeted interventions.

Despite this urgency, empirical evidence linking household food insecurity and upstream structural determinants to child undernutrition in migrant populations along the Thailand–Myanmar border remains limited. Existing studies often rely on simplistic bivariate analyses, focusing narrowly on short-term hunger or dietary diversity while overlooking the complex, cumulative effects of prolonged food insecurity. Furthermore, region-specific research within Myanmar frequently fails to capture the unique macro-structural, environmental, and legal challenges faced by transient migrant households in border settlements. Consequently, a critical knowledge gap persists regarding how upstream structural determinants cascade through underlying household-level factors to ultimately drive individual anthropometric outcomes in this complex border environment. To address these gaps, this study narrows its focus to test a comprehensive structural equation model (SEM) based on the integrated UNICEF-FNS framework. Specifically, this study examines both the direct and indirect pathways of household food insecurity as it impacts child undernutrition, with the indirect effects mediated through child dietary intake and morbidity factors, alongside the overarching multi-level relationships driving stunting, wasting, and underweight among children under five in migrant households along the Thailand–Myanmar border.

## 2 Methods

### 2.1 Ethics Statement

Ethical approval for this study was granted by the Ethical Review Committee of the Faculty of Graduate Studies, Mahidol University (Ref: MU-CIRB 2025/157.0805) and the Community Ethics Advisory Board (Ref: CEAB-2025–009), which oversees research involving migrant communities along the Thailand–Myanmar border. Administrative clearances were obtained from local organizations currently operating in the Mae Sot, Phop Phra, and Sop Moei districts. Community leaders were informed about the study’s purpose and survey dates several days in advance to foster trust and ensure transparency.

Participant recruitment and data collection were conducted from 15/06/2025–15/07/2025. Upon arrival at the survey sites, mothers of eligible children (aged 6–59 months) were provided with a translated information sheet in Burmese. All participating mothers were adult guardians who demonstrated full capacity to understand the study procedures and provide independent informed consent during the pre-interview briefing; thus, formal capacity assessments were not required. Due to varying literacy levels and the transient nature of this migrant population, informed verbal consent was obtained from the mothers, acting as legal guardians for their minor children, prior to any interviews or anthropometric measurements. This specific verbal consent procedure was explicitly reviewed and approved by both ethics committees, with the MU-CIRB operating in full compliance with international guidelines for human research protection. To ensure accountability, the verbal consent process was witnessed by three trained research assistants and community volunteers at each respective location. Consent was formally documented via a dedicated, mandatory verification checkbox within the Kobo Toolbox digital data collection system before the survey instrument could proceed. Participant confidentiality was strictly maintained through the use of unique identification codes, and data were stored securely, accessible only to the primary research team.

### 2.2 Study design and settings

This community-based cross-sectional study was conducted in rural areas of Tak and Mae Hong Son provinces along the Thailand–Myanmar border. These regions were purposively selected due to the high documented prevalence of childhood stunting and wasting [15]. Tak Province, bordering Myanmar’s Kayin State, serves as a primary hub for cross-border migration, with the Phop Phra and Mae Sot districts hosting substantial migrant and refugee populations. In contrast, Mae Hong Son Province which borders Myanmar’s Shan, Kayah, and Kayin States, has a long history of accommodating conflict-affected populations. Within this province, the Sop Moei district was specifically included to represent particularly remote and underserved communities. Collectively, these sites were selected to capture the diverse socioeconomic and environmental challenges faced by vulnerable migrant households across the border zone.

**Fig 1:**
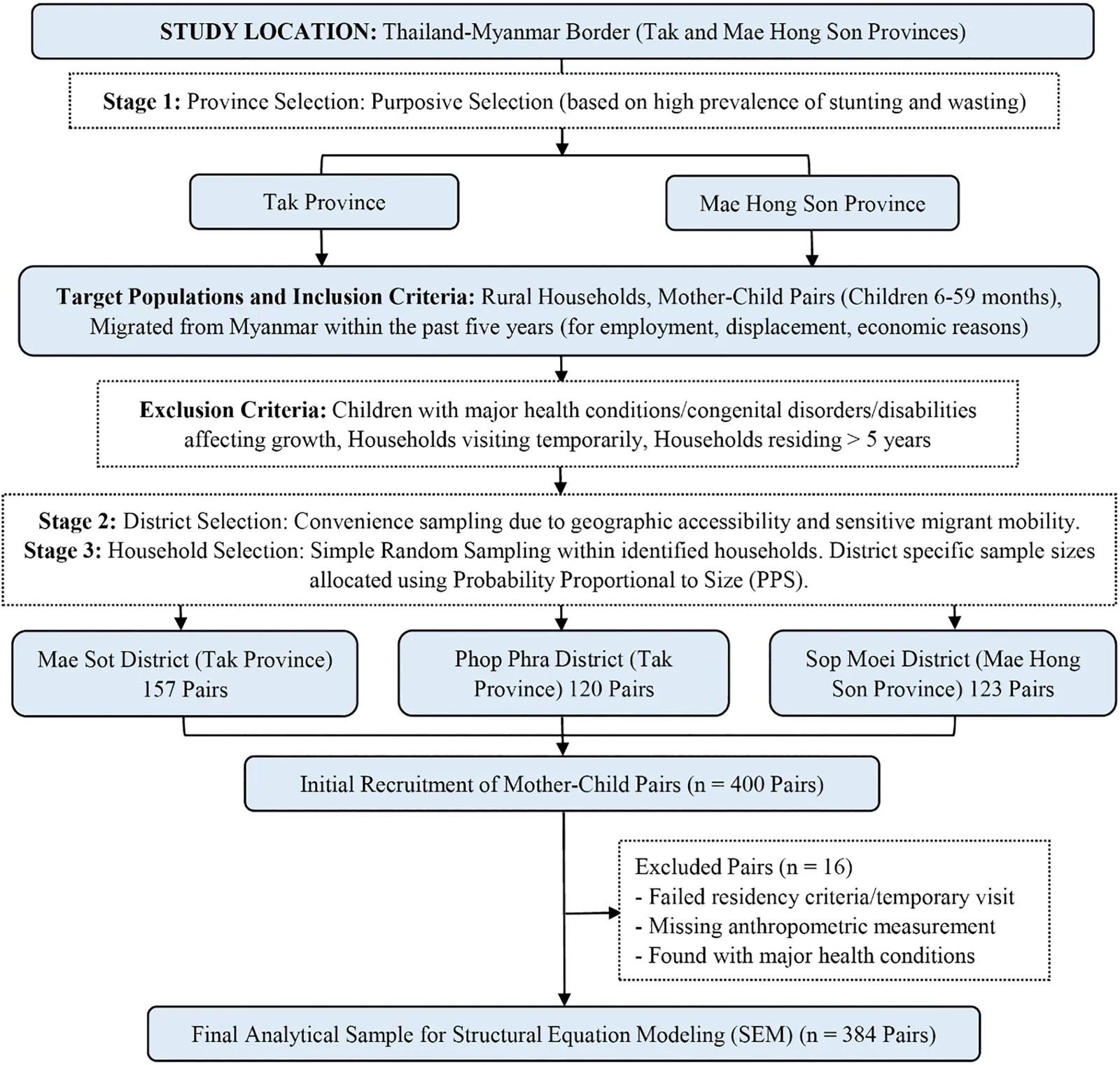
Multistage sampling framework and participant selection flow diagram.

As detailed in Fig 1, a multi-stage sampling design was employed. Stage 1 involved the purposive selection of Tak and Mae Hong Son provinces based on high historical prevalences of undernutrition. In Stage 2, Mae Sot, Phop Phra, and Sop Moei districts were selected via convenience sampling due to geographic accessibility and sensitive migrant mobility constraints. District-specific sample sizes were allocated using probability proportional to size (PPS), targeting an initial recruitment of 400 mother-child pairs: 157 pairs from Mae Sot, 120 pairs from Phop Phra, and 123 pairs from Sop Moei. In Stage 3, individual households were selected through simple random sampling. Since official state-level census records were unavailable in these border zones, localized household registries updated by village tract leaders and community-based organizations served as the definitive sampling frame. Eligible households on these rosters were assigned sequential identification numbers, and random selection was executed using a computer-generated random number table until each district's PPS quota was achieved. In households with multiple eligible pairs, exactly one mother-child pair was randomly selected via an on-site lottery method to eliminate intra-household clustering bias. Eligible participants included mother-child pairs (children aged 6–59 months) who had migrated from Myanmar within the past five years for employment, displacement, or economic reasons. Exclusion criteria consisted of children with major health conditions or disabilities affecting growth, as well as households visiting temporarily or residing in the area for more than five years.

The required sample size was calculated using Cochran’s (1977) formula (n = Z^2^pq/e^2^). Based on a 95% confidence level and a 5% margin of error, the final analytical sample was determined to be 384. Although 400 pairs were initially recruited to buffer against potential data incompleteness, 16 pairs were excluded due to missing anthropometric measurements or residency criteria. A sensitivity check confirmed these exclusions were random, resulting in a final sample of 384 pairs for structural equation modeling (SEM).

### 2.3 Research instruments

Data was collected using a structured questionnaire covering seven domains: maternal and child factors, health services, feeding and dietary practices, household and environmental contexts, socioeconomic determinants, household food insecurity, and anthropometric measurements.

#### 2.3.1 Anthropometric assessment.

Anthropometric measurements were obtained for all children aged 6–59 months following standard WHO protocols. Weight was measured with minimal clothing using a calibrated scale accurate to 0.1 kg. If a child refused to be weighed alone, the mother was weighed with and without the child, and the child’s weight was calculated by subtraction. To eliminate systematic measurement errors in height, age-appropriate positioning was strictly implemented according to WHO guidelines. Chronological age served as the definitive criterion for measurement positioning: for children aged 24–59 months, standing height was measured without footwear using a standard height board accurate to 0.1 cm, ensuring five contact points (heels, calves, buttocks, shoulders, and back of the head) touched the vertical board. Conversely, for children under 24 months of age (6–23 months), recumbent length was measured using the same board placed horizontally on a flat surface [17]. The nutritional status of children under five was assessed using WHO Anthro software (version 3.2.2). The nutritional status of children under five was assessed using WHO Anthro software (version 3.2.2). During data entry, the exact measurement position (recumbent vs. standing) was strictly verified against chronological age and specified for each child, allowing the software to automatically apply the standard 0.7 cm correction factor where deviations occurred, thereby guaranteeing mathematical accuracy. Growth outcomes were evaluated based on Z-scores for linear growth (height-for-age, HAZ), acute growth (weight-for-height, WHZ), and overall nutritional status (weight-for-age, WAZ) [18,19]. The cut-off for each undernutrition indicator was set at below -2 standard deviations (SD) from the WHO Child Growth Standards median [20,21].

#### 2.3.2 Household food insecurity assessment.

Household food insecurity was assessed using the Household Food Insecurity Access Scale (HFIAS), specifically adapted for households with children under five. Caregivers reported their household’s experience of nine food access conditions over the previous 30 days, with response options: “Never” (0), “Rarely” (1–2 times), “Sometimes” (3–10 times), and “Often” (> 10 times). Based on the HFIAS responses, households were classified into four standardized categories reflecting the severity of food insecurity: food secure, mildly food insecure, moderately food insecure, and severely food insecure. Classification into these four categories was determined by the household's responses to the nine indicators, using the established decision rules based on the most severe condition reported. This methodology follows the guidelines outlined by the Food and Nutrition Technical Assistance III Project (FANTA) [22].

#### 2.3.3 Morbidity assessment.

Child morbidity was assessed via 14-day maternal recall for diarrhea, fever, and acute respiratory infection (ARI) [23,24]. Diarrhea was defined as > 3 loose stools within 24 hours; fever was elevated body temperature (> 38°C/100.4°F); and ARI was defined as a sudden onset of fever accompanied by respiratory symptom, such as cough or sore throat, or difficulty breathing.

#### 2.3.4 Minimum meal frequency (MMF).

Minimum Meal Frequency was assessed to determine if a child consumed a sufficient number of meals in the preceding 24 hours, with criteria defined by standard WHO/UNICEF IYCF guidelines and stratified by breastfeeding status for children aged 6–23 months. For breastfed infants, the threshold was set at ≥ 2 times for 6–8 months and ≥ 3 times for 9–23 months, while for non-breastfed children (6–23 months), the threshold was set at ≥ 4 times. Conversely, for older children aged 24–59 months who had already transitioned to regular family foods, adequate intake was evaluated using standard daily meal frequency, defined as consuming at least three main meals alongside nutritious snacks within the prior 24 hours to fulfill age-specific baseline energy and caloric requirements [25,26].

#### 2.3.5 Minimum dietary diversity (MDD).

Minimum Dietary Diversity was assessed via a 24-hour maternal recall method to capture structural dietary quality across the entire 6–59 months target population. In strict alignment with the baseline WHO (2010) and FAO (2013) core guidelines for younger cohorts, alongside standard dietary diversity matrices for older under-five children, a standardized 7-food-group framework was utilized [25,27,28]. The evaluated food groups comprised: (1) grains, roots, and tubers; (2) legumes and nuts; (3) dairy products; (4) flesh foods; (5) eggs; (6) vitamin A-rich fruits and vegetables; and (7) other fruits and vegetables. Consuming ≥ 4 out of these seven distinct food groups within the prior 24 hours was classified as “adequate” dietary diversity for all evaluated age groups.

### 2.4 Theoretical foundation and framework

This study is grounded in an integrated framework combining the UNICEF Conceptual Framework of the Determinants of Maternal and Child Nutrition [6,7] and Food and Nutrition Security (FNS) Theory [29]. This model systematically categorizes multi-level determinants of undernutrition in children under five via a causal hierarchy. At the most proximal level, immediate determinants directly affecting individual child nutritional status include disease occurrence (diarrhea, fever, and ARI) and infant and young child feeding (IYCF) practices. To address the biological mechanisms within this layer, the interaction between recent illness and dietary practices is interpreted through the Malnutrition-Infection Synergistic Cycle, accounting for how environmental pathogens can drive subclinical gut inflammation (Environmental Enteric Dysfunction) to impair nutrient utilization. These immediate factors are modulated by underlying determinants at the household and community level, aligned with FNS pillars: economic access (household food insecurity, income, and employment) and utilization/care (delivery location, attendant profession, and sanitation practices). Within this underlying layer, the critical link between economic distress and child dietary quality is understood via household economic coping behaviors under duress. This mechanism draws on consumer substitution principles, where financially constrained households under duress are structurally forced to prioritize cheap caloric satiety over micronutrient density. This economic trade-off directly operationalizes how resource constraints within the household systematically suppress the dietary diversity required for child growth. The most distal enabling determinants define the macro-structural resource environment, including household composition and water access stability. In this study, this structural layer is contextually expanded to include the length of migration, modeling how transient migration shocks and recent displacement disrupt social capital and access to border safety nets. This structured approach models the complex, cascading pathways from structural enabling causes through underlying and immediate factors to absolute anthropometric outcomes (stunting, wasting, and underweight).

### 2.5 Variable operationalization for structural equation modeling (SEM)

**Table 1** delineates the operational definitions, coding schemes, and classifications of the variables incorporated into the Structural Equation Model (SEM).

**Table 1:** Operationalization of Study variables for SEM.

Variable	Definition	Code/Measurement	Type	Level
Number of U5 Children	Count of children under age 5	Numeric value	Continuous	Basic factors
Water enough in past month (WE)	Water availability and reliability	0 (Strongly Disagree) to 4 (Strongly Agree)	Ordinal	
Water access in dry season (WA)	Water availability and reliability	0 (Strongly Disagree) to 4 (Strongly Agree)	Ordinal	
Water access in entire year (WY)	Water availability and reliability	0 (Strongly Disagree) to 4 (Strongly Agree)	Ordinal	
Household food insecurity status (HFIS)	HFI categories (severity)	0 = Severe, 1 = Moderate, 2 = Mild, 3 = Secure	Ordinal	Underlying factors
Employment status (ES)	Job status of household head	0 = Informal (Unstable), 1 = Formal (Stable)	Binary	
Monthly income (IC)	Threshold based on poverty line	0 = ≤7,304 Baht, 1 = > 7,304 Baht	Binary	
Delivered place (ID)	Place of delivery (Institutional delivery)	0 = Outside health facility, 1 = At health facility	Binary	
Delivered by (DB)	Attendant’s profession during delivery	0 = Non-health professional, 1 = Health professional	Binary	
Safety Sanitation practices	JMP classification	0 = Unsafely managed, 1 = Safely managed	Binary	
MMF	Minimum Meal Frequency (WHO IYCF indicator)	0 = Not met, 1 = Met	Binary	Immediate factors
MDD	Minimum Dietary Diversity (WHO IYCF indicator)	0 = Not met, 1 = Met	Binary	
Diarrhea	Diarrhea past 2 weeks	0 = No, 1 = Yes	Binary	
Fever	Fever past 2 weeks	0 = No, 1 = Yes	Binary	
ARI	ARI past 2 weeks	0 = No, 1 = Yes	Binary	
HAZ	Height-for-age Z-score	Numeric Z-score value	Continuous	Outcome
WHZ	Weight-for-height Z-score	Numeric Z-score value	Continuous	
WAZ	Weight-for-age Z-score	Numeric Z-score value	Continuous	

HFIS, Household Food Insecurity (Severity); ARI, Acute Respiratory Infection; JMP, Joint Monitoring Programme; IYCF, Infant and Young Child Feeding.

### 2.6 Data management and analysis

Data management and statistical analyses were conducted using IBM SPSS Statistics (Version 29). Anthropometric Z-scores (HAZ, WHZ, and WAZ) were calculated via WHO Anthro software (v.3.2.2) based on the 2006 WHO Child Growth Standards. Prior to analysis, the dataset was screened for missing values and inconsistencies. Data quality was ensured through biological plausibility checks, including the cross-verification of child age and biological sex to maintain the accuracy of anthropometric computations. Descriptive statistics were used to summarize the study population; continuous data were presented as medians and interquartile ranges (IQRs), while categorical variables were reported as frequencies and percentages. Chi-square tests of independence were conducted to explore bivariate associations between independent variables and undernutrition outcomes.

For Structural Equation Modeling (SEM), variables were categorized by their theoretical roles: basic, underlying, immediate, and nutritional outcomes. To ensure directional consistency, Household Food Insecurity (HFI) was coded in ascending order (0 = severely food insecure to 3 = food secure), where higher scores represent favorable food security. Consequently, higher HFI scores align with improved socioeconomic indicators, dietary diversity (MDD), and feeding frequency (MMF). Conversely, morbidity was operationalized as binary (1 = illness occurrence). Anthropometric outcomes were maintained as continuous Z-scores (HAZ, WHZ, and WAZ) to maximize statistical power, while binary categories (stunting, wasting, and underweight) were reserved for descriptive prevalence reporting. Full operational definitions are provided in **Table 1**.

The analysis followed a two-step approach. First, the measurement model was validated using Confirmatory Factor Analysis (CFA) to evaluate construct validity and reliability [30]. Standardized factor loadings (> 0.70), Average Variance Extracted (AVE ≥ 0.50), and Composite Reliability (CR ≥ 0.70) were examined [31]. Second, SEM was performed in IBM SPSS AMOS (v. 29) to test hypothesized direct and indirect pathways. Separate path models were specified for each of the three continuous outcomes. Given the multivariate non-normality of the data, model estimation was performed using Maximum Likelihood (ML) with bias-corrected bootstrapping (2,000 resamples). This approach was employed to provide robust standard errors and 95% confidence intervals, particularly for the accurate estimation of indirect effects. Categorical indicators were treated as observed variables and incorporated into the models using appropriate ordinal or dummy coding.

Overall model fit was assessed using established indices (CFI, NFI, TLI ≥ 0.90; RMSEA, SRMR ≤ 0.08) [31]. The proportion of explained variance (R^2^) was reported for each endogenous outcome to evaluate the model's explanatory power. Finally, a sensitivity analysis was conducted using multiple linear regression to validate the robustness of the primary findings. Continuous HFIAS scores (0–27) were utilized as predictors for HAZ, WHZ, and WAZ.

### 2.7 Data quality and instrument validation

#### 2.7.1 Quality assurance and reliability.

Several measures were implemented to ensure data quality and minimize recall and linguistic biases. Enumerators received intensive training on standardized probing for 14-day morbidity recall, 24-hour feeding practices, and anthropometric measurement. The survey instrument was professionally translated and back-translated between English and Burmese to maintain conceptual accuracy. During fieldwork, the principal investigator conducted random on-the-spot reviews, and all forms underwent a daily comprehensive review for consistency and adherence to the study protocol.

Instrument reliability was established via a two-step procedure involving a pilot test (n = 30) to refine the dual-language versions. Internal consistency for the final scales was confirmed using Cronbach’s alpha (α), with coefficients for household food insecurity (α = 0.857) and water availability (α = 0.859) exceeding the 0.70 threshold.

#### 2.7.2 Measurement model: Validity and correlation analysis.

The measurement model was evaluated for validity and reliability through Confirmatory Factor Analysis (CFA). As detailed in **Table A in** **S1 Appendix****,** indicator reliability was confirmed with standardized factor loadings exceeding 0.70. Construct reliability was established with high Composite Reliability (CR) values for socioeconomic status (CR = 0.943), healthcare services (CR = 0.989), and water availability (CR = 0.906), all well above the 0.70 benchmark. Content validation was verified by three independent experts using the Item-Objective Congruence (IOC) index (> 0.60).

Convergent validity was confirmed as the Average Variance Extracted (AVE) for socioeconomic status (0.650), healthcare services (0.782), and water availability (0.740) all exceeded the 0.50 criterion. Discriminant validity was verified using the Fornell-Larcker criterion (**Table B in** **S1 Appendix**); diagonal elements, representing the square root of the AVE for socioeconomic status (0.806), healthcare services (0.884), and water availability (0.860), consistently exceeded all off-diagonal inter-construct correlations.

Finally, latent construct correlations were evaluated across the HAZ, WHZ, and WAZ structural models (**Table C in** **S1 Appendix**). Internal associations remained consistent; notably, socioeconomic status (SES) showed strong positive associations with healthcare services (r: .257–.263, p < .001), while water availability strongly correlated with safety and sanitation practices, reaching r = .308 (p < .001) in the WHZ model and r = .318 (p < .001) in both the HAZ and WAZ models. Crucially, within the WHZ model, water availability demonstrated a significant negative correlation with the length of migration (r = -.151, p = .003), while a distinct negative association also emerged between minimum meal frequency (MMF) and minimum dietary diversity (MDD) (r = -.140, p = .007). Significant morbidity clusters emerged between diarrhea and fever in the WHZ model (r = .315, p < .001) and diarrhea and acute respiratory infection in the WAZ model (r = .148, p = .005), suggesting shared vulnerability to childhood illnesses. All correlation coefficients remained below 0.85, further confirming sufficient discriminant validity and the absence of significant conceptual overlap.

## 3 Results

The study assessed 384 mother-child pairs with children aged 6–59 months. Overall, more than one-third of the children (37.0%) experienced at least one form of undernutrition. The overall prevalence of stunting, wasting, and underweight was 18.0%, 19.8%, and 18.5%, respectively. Descriptive comparisons by sex showed that the prevalence of stunting was slightly higher in males (19.4%) than in females (16.4%). Conversely, females exhibited higher observed rates of wasting (20.8% vs. 18.9%) and underweight status (20.8% vs. 16.4%) compared to their male counterparts.

### 3.1 Bivariate associations with child undernutrition outcomes

The characteristics of the study population and the results of the bivariate analysis using Chi-square (χ²) tests to identify factors associated with stunting, wasting, and underweight are summarized in **Table 2**. Stunting was significantly associated with indicators of early life care and household economic stability. Significant associations were found with employment status (p < .001) and monthly income (p < .001), with 91.3% of stunted children originating from households with informal/unstable employment. Regarding birth and healthcare, stunting was significantly linked to low birth weight (p = .028), delivery outside a health facility (p = .002), delivery by non-health professionals (p < .001), and incomplete routine immunization (p < .001). Nutritional and health mediators also played a significant role; minimum dietary diversity (p = .002) and household food insecurity (p < .001) were both strong predictors, with 39.1% of stunted children living in severely food-insecure households. Detailed analysis of specific dietary contributions revealed a heavy reliance on staples; grains, roots, tubers, and plantains were the most frequently consumed (92.7%), followed by flesh foods (82.3%). In contrast, the consumption of nutrient-dense pulses, nuts, and seeds was notably low (58.9%), indicating a critical gap in protein and micronutrient variety (**Table D in** **S1 Appendix**). Furthermore, recent illness (p = .002) was significantly associated with stunting, affecting two-thirds of the stunted population.

**Table 2:** Characteristics of the study population and their associations with child undernutrition (n = 384).

Independent Variables	Total (n = 384) n (%)	Stunting (n = 69) n (%)	p-value	Wasting (n = 76) n (%)	p-value	Underweight (n = 71) n (%)	p-value
Mother’s education			.157		.046*		.491
Low education	160 (41.7)	34 (49.3)		24 (31.6)		27 (38.0)	
High education	224 (58.3)	35 (50.7)		52 (68.4)		44 (62.0)	
Number of under 5 children			.118		.684		.070
1 child	329 (85.7)	55 (79.7)		64 (84.2)		56 (78.9)	
≥2 children	55 (14.3)	14 (20.3)		12 (15.8)		15 (21.1)	
Employment status			<.001***		.006**		<.001***
Informal (Unstable)	246 (64.1)	63 (91.3)		59 (77.6)		63 (88.7)	
Formal (Stable)	138 (35.9)	6 (8.7)		17 (22.4)		8 (11.3)	
Monthly income			<.001***		<.001***		<.001***
≤7,304 Baht	261 (68.0)	68 (98.6)		65 (85.5)		66 (93.0)	
>7,304 Baht	123 (32.0)	1 (1.4)		11 (14.5)		5 (7.0)	
Length of migration			.248		.030*		.232
<1 year	42 (10.9)	4 (5.8)		12 (15.8)		11 (15.5)	
1-3 years	150 (39.1)	26 (37.7)		36 (47.4)		30 (42.3)	
3-5 years	192 (50.0)	39 (56.5)		28 (36.8)		30 (42.3)	
Low birth weight			.028*		.587		.003**
Yes (<2.5 kg)	39 (10.2)	12 (17.4)		9 (11.8)		14 (19.7)	
No (≥2.5 kg)	345 (89.8)	57 (82.6)		67 (88.2)		57 (80.3)	
Preterm birth			.381		.164		.037*
Yes (<37 weeks)	39 (10.2)	9 (13.0)		11 (14.5)		12 (16.9)	
No (≥37 weeks)	345 (89.8)	60 (87.0)		65 (85.5)		59 (83.1)	
Delivered place			.002**		.633		.107
Outside health facility	35 (9.1)	13 (18.8)		8 (10.5)		10 (14.1)	
At Health facility	349 (90.9)	56 (81.2)		68 (89.5)		61 (85.9)	
Delivered by			<.001***		.663		.007**
Non-health professional	26 (6.8)	12 (17.4)		6 (7.9)		10 (14.1)	
Health professional	358 (93.2)	57 (82.6)		70 (92.1)		61 (85.9)	
Routine immunization			<.001***		.769		.112
Partially/no vaccination	101 (26.3)	29 (42.0)		21 (27.6)		24 (33.8)	
Fully vaccinated	283 (73.7)	40 (58.0)		55 (72.4)		47 (66.2)	
Disease past 2 weeks			.002**		<.001***		<.001***
Yes	191 (49.7)	46 (66.7)		52 (68.4)		54 (76.1)	
No	193 (50.3)	23 (33.3)		24 (31.6)		17 (23.9)	
Safety sanitation practices			.855		.892		.888
Unsafety managed	230 (59.9)	42 (60.9)		45 (59.2)		42 (59.2)	
Safety managed	154 (40.1)	27 (39.1)		31 (40.8)		29 (40.8)	
Water availability level			.329		.726		.243
Low/Moderate	82 (21.4)	18 (26.1)		15 (19.7)		19 (26.8)	
High	302 (78.6)	51 (73.9)		61 (80.3)		52 (73.2)	
Minimum meal frequency			.174		.177		.114
Not meet	221 (54.9)	43 (62.3)		47 (61.8)		45 (63.4)	
Meet	173 (45.1)	26 (37.7)		29 (16.8)		26 (36.6)	
Minimum diet diversity			.002**		.146		.096
Not meet	90 (23.4)	26 (37.7)		13 (17.1)		22 (31.0)	
Meet	294 (76.6)	43 (62.3)		63 (82.9)		49 (69.0)	
Food insecurity level			<.001***		.086		.020*
Severely food insecure	94 (24.5)	27 (39.1)		14 (18.4)		24 (33.8)	
Moderately food insecure	80 (20.8)	19 (27.5)		18 (23.7)		20 (28.2)	
Mildly food insecure	71 (18.5)	17 (24.6)		9 (11.8)		10 (14.1)	
Food secure	139 (36.2)	6 (8.7)		35 (46.1)		17 (23.9)	

Note: n is frequency, p-values were determined using Chi-square (χ²) tests. Statistical significance is indicated as * p < .05, ** p < .01, *** p < .001.

Wasting was significantly associated with variables reflecting immediate economic stability and morbidity. Higher levels of wasting were linked to mother’s education (p = .046) and length of migration (p = .030). Consistent with other indicators, employment status (p = .006) and monthly income (p < .001) remained significant determinants. While birth history and nutrition access (dietary diversity and food security) did not reach statistical significance for wasting (p > .05), recent illness showed an extremely strong association (p < .001). Nearly 70% of children identified as wasted had suffered from an illness in the two weeks preceding the survey, highlighting the acute impact of disease on weight loss.

Underweight status reflected a combination of both chronic birth outcomes and acute health shocks. Significant socioeconomic associations were found for employment status (p < .001) and monthly income (p < .001). Birth history was particularly influential for this indicator, with low birth weight (p = .003), preterm birth (p = .037), and delivery by non-health professionals (p = .007) all showing significant associations. In terms of nutrition and morbidity, household food insecurity was significantly linked to being underweight (p = .020). Furthermore, recent illness showed the strongest correlation with this outcome (p < .001), with 76.1% of underweight children having been ill recently. Environmental factors, such as sanitation and water availability, did not show significant associations with underweight status in this analysis.

### 3.2 Structural equation modeling: Measurement and structural fit

The goodness-of-fit indices for the measurement model and the three structural models (HAZ, WHZ, and WAZ) are summarized in **Table 3**. Overall, the results indicated excellent fit across all models, with non-significant χ² values (p > .05) suggesting no significant discrepancy between the hypothesized models and observed data. All incremental fit indices (NFI, CFI, TLI) consistently exceeded the 0.95 threshold, while absolute fit indices (RMSEA, SRMR) remained within the “excellent” range, confirming statistical robustness. Specifically, the measurement model achieved a near-perfect fit (χ²/df = 0.908, p = .538, CFI = 1.000, RMSEA = 0.000). Similar high-quality fits were observed for the HAZ (χ²/df = 1.061, p = .356, CFI = 0.998), WHZ (χ²/df = 1.204, p = .096, CFI = 0.989), and WAZ models (χ²/df = 1.013, p = .448, CFI = 0.999), with all RMSEA values < 0.026. These results indicate that the latent constructs are measured with high precision and the model-implied covariance matrices align closely with observed data.

**Table 3:** Goodness-of-fit indices for measurement and structural models.

Fit Index	CFA Model	HAZ Model	WHZ Model	WAZ Model	Recommended Threshold
χ²/df	0.908	1.061	1.204	1.013	< 3.0 (Good)
P-value	0.538	0.356	0.096	0.448	> .05 (Good)
NFI	0.992	0.965	0.941	0.962	≥ 0.95 (Excellent), ≥ 0.90 (Good)
CFI	1.000	0.998	0.989	0.999	≥ 0.95 (Excellent), ≥ 0.90 (Good)
TLI	1.000	0.997	0.985	0.999	≥ 0.95 (Excellent), ≥ 0.90 (Good)
RMSEA	0.000	0.013	0.023	0.006	< 0.05 (Excellent), < 0.08 (Acceptable)
RMSEA 90% CI	[0.000, 0.048]	[0.000, 0.036]	[0.000, 0.038]	[0.000, 0.031]	Upper bound < 0.08 (Excellent)
PCLOSE	0.959	0.999	0.999	1.000	> 0.05 (Excellent)
RMR/SRMR	0.008	0.018	0.030	0.022	< 0.05 (Excellent)
GFI	0.992	0.979	0.967	0.977	≥ 0.90 (Excellent)
AGFI	0.981	0.963	0.948	0.962	≥ 0.90 (Excellent)

Note: χ²/df, Chi-square divided by degrees of freedom; NFI, Normed Fit Index; CFI, Comparative Fit Index; TLI, Tucker-Lewis Index; RMSEA, Root Mean Square Error of Approximation; RMSEA 90% CI, 90% Confidence Interval for RMSEA; PCLOSE, P-value for close fit; RMR/SRMR, Root Mean Square Residual/Standardized Root Mean Square Residual; GFI, Goodness of Fit Index; AGFI, Adjusted Goodness of Fit Index.

The results of the structural equation models are summarized in **Table 4**, which presents the standardized path coefficients (β) and their respective levels of significance for the predictors linear growth (HAZ), acute growth (WHZ), and weight-for-age (WAZ).

**Table 4:** Standardized path coefficients from structural equation model of undernutrition (n = 384).

Path	Std Estimate (β)	Robust S.E.	95% CI	p-value
**A: Linear Growth (HAZ) Model**				
Water Availability -- > HFI Category	0.131	0.057	(0.023, 0.244)	.020
Safety Sanitation Practice -- > HFI Category	0.181	0.05	(0.081, 0.282)	.001
Socioeconomic Status -- > HFI Category	0.311	0.055	(0.204, 0.416)	.001
Number of U 5 Children -- > HFI Category	-0.159	0.046	(-0.249, -0.069)	.003
Healthcare Services -- > MDD	0.167	0.065	(0.049, 0.299)	.007
HFI Category -- > MDD	0.183	0.048	(0.085, 0.272)	.001
MDD -- > Diarrhea	-0.121	0.055	(-0.013, -0.229)	.026
Socioeconomic Status -- > Diarrhea	-0.179	0.05	(-0.075, -0.274)	.002
Socioeconomic Status -- > Monthly Income	0.837	0.048	(0.746, 0.936)	.001
Socioeconomic Status -- > Employment Status	0.775	0.050	(0.670, 0.866)	.002
Water Availability -- > Water Enough Past Month	0.833	0.036	(0.745, 0.889)	.002
Water Availability -- > Water Access Dry Season	0.780	0.032	(0.712, 0.838)	.001
Water Availability -- > Water Access Entire Year	0.833	0.036	(0.745, 0.889)	.002
Healthcare Services -- > Delivered By	0.894	0.076	(0.734, 1.026)	.001
Healthcare Services -- > Delivered Place	0.871	0.073	(0.709, 0.996)	.002
Socioeconomic Status -- > HAZ	0.230	0.054	(0.125, 0.336)	.001
HFI Category -- > HAZ	0.113	0.052	(0.006, 0.212)	.033
Diarrhea -- > HAZ	-0.109	0.050	(-0.012, -0.204)	.028
**B: Acute Growth (WHZ) Model**				
Water Availability -- > HFI Category	0.131	0.057	(0.023, 0.243)	.019
Safety Sanitation Practice -- > HFI Category	0.179	0.050	(0.080, 0.281)	.001
Number of U 5 Children -- > HFI Category	-0.158	0.046	(-0.248, -0.069)	.003
Socioeconomic Status -- > HFI Category	0.313	0.054	(0.206, 0.419)	.001
Healthcare Services -- > MDD	0.152	0.067	(0.030, 0.287)	.015
HFI Category -- > MDD	0.190	0.048	(0.094, 0.280)	.001
Water Availability -- > MMF	0.294	0.048	(0.200, 0.384)	.001
Socioeconomic Status -- > Diarrhea	-0.177	0.051	(-0.274, -0.073)	.002
MDD -- > Diarrhea	-0.125	0.051	(-0.224, -0.025)	.015
HFI Category -- > Fever	-0.125	0.052	(-0.226, -0.019)	.020
Socioeconomic Status -- > Fever	-0.219	0.051	(-0.315, -0.116)	.002
Socioeconomic Status -- > Monthly Income	0.821	0.047	(0.731, 0.917)	.001
Socioeconomic Status -- > Employment Status	0.792	0.049	(0.693, 0.885)	.001
Water Availability -- > Water Enough Past Month	0.782	0.032	(0.712, 0.838)	.002
Water Availability -- > Water Access Dry Season	0.833	0.036	(0.744, 0.888)	.002
Water Availability -- > Water Access Entire Year	0.957	0.016	(0.920, 0.985)	.002
Healthcare Services -- > Delivered By	0.901	0.078	(0.729, 1.035)	.002
Healthcare Services -- > Delivered Place	0.865	0.075	(0.698, 0.991)	.002
Length of migration -- > MMF	0.127	0.049	(0.027, 0.220)	.013
Fever -- > Wasting	-0.159	0.050	(-0.262, -0.066)	.003
Diarrhea -- > Wasting	-0.125	0.063	(-0.243, 0.001)	.049
MMF -- > Wasting	0.107	0.047	(0.011, 0.198)	.035
Length of migration -- > Wasting	0.155	0.045	(0.060, 0.237)	.001
**C: Weight-for-Age (WAZ) Model**				
Water Availability -- > HFI Category	0.127	0.057	(0.019, 0.241)	.023
Safety Sanitation Practice -- > HFI Category	0.180	0.050	(0.080, 0.281)	.001
Number of U 5 Children -- > HFI Category	-0.155	0.046	(-0.247, -0.067)	.002
Socioeconomic Status -- > HFI Category	0.318	0.054	(0.210, 0.420)	.001
Healthcare Services -- > MDD	0.167	0.065	(0.048, 0.299)	.007
HFI Category -- > MDD	0.183	0.048	(0.085, 0.272)	.001
Socioeconomic Status -- > Diarrhea	-0.180	0.051	(-0.078, -0.279)	.002
MDD -- > Diarrhea	-0.112	0.055	(-0.010, -0.222)	.034
Socioeconomic Status -- > ARI	-0.175	0.054	(-0.077, -0.291)	.001
Socioeconomic Status -- > Monthly Income	0.818	0.042	(0.736, 0.903)	.001
Socioeconomic Status -- > Employment Status	0.790	0.044	(0.702, 0.876)	.001
Water Availability -- > Water Enough Past Month	0.833	0.036	(0.744, 0.889)	.002
Water Availability -- > Water Access Dry Season	0.780	0.032	(0.712, 0.838)	.001
Water Availability -- > Water Access Entire Year	0.959	0.016	(0.923, 0.987)	.001
Healthcare Services -- > Delivered By	0.896	0.076	(0.736, 1.028)	.001
Healthcare Services -- > Delivered Place	0.869	0.072	(0.707, 0.996)	.002
Socioeconomic Status -- > WAZ	0.294	0.051	(0.188, 0.388)	.001
Diarrhea -- > WAZ	-0.217	0.045	(-0.123, -0.304)	.001
ARI -- > WAZ	-0.144	0.055	(-0.040, -0.249)	.014

#### 3.2.1 Linear growth (HAZ) model.

The standardized path coefficients for the linear growth model reveal several significant relationships. Socioeconomic status had the strongest positive direct effect on household food insecurity (HFI) category (β = 0.311, p < .001), followed by safety sanitation practice (β = 0.181, p = .001) and water availability (β = 0.131, p = .020). This suggested that better socioeconomic status, safer sanitation practices, and greater water availability were associated with improved food security. Conversely, having a greater number of children under five was associated with lower household food insecurity (β = -0.159, p = .003).

The relationships between socioeconomic status and its indicators, monthly income (β = 0.837, p = .001) and employment status (β = 0.775, p = .002), were very strong, confirming that higher socioeconomic status was highly predictive of better financial and employment conditions. Similarly, water availability had a powerful positive influence on all aspects of water access: water enough past month (β = 0.833, p = .002), water access dry season (β = 0.780, p = .001), and water access entire year (β = 0.833, p = .002). Healthcare services had a strong positive association with their indicators, delivered by (β = 0.894, p = .001) and delivered place (β = 0.871, p = .002), indicating that the quality of healthcare was strongly linked to the type of provider and location.

Both HFI category (β = 0.183, p = .001) and healthcare services (β = 0.167, p = .007) had a positive impact on minimum dietary diversity (MDD). This suggested that food security and access to healthcare were key predictors of a child's nutritional intake. Finally, the model showed that socioeconomic status (β = 0.230, p = .001) and HFI category (β = 0.113, p = .033) had a positive relationship with linear growth (HAZ). This indicated that better socioeconomic conditions and improved food security were linked to enhanced linear growth outcomes in children. Conversely, the path from diarrhea to linear growth (HAZ) was negative (β = -0.109, p = .028), indicating that the occurrence of diarrhea was associated with stunted linear growth (lower HAZ scores).

#### 3.2.2 Acute growth (WHZ) model.

The standardized path coefficients of the acute growth model show several factors with significant positive influences. Socioeconomic status had the strongest positive effect on household food insecurity (HFI) category (β = 0.313, p = .001), indicating that a higher socioeconomic status is linked to less food insecurity. The association between socioeconomic status and both monthly income (β = 0.821, p = .001) and employment status (β = 0.792, p = .001) was very strong, suggesting that socioeconomic well-being is highly predictive of better financial and employment conditions. Water availability had a very strong positive influence on all aspects of water access. The path to water access entire year was the strongest (β = 0.957, p = .002), followed by water enough past month (β = 0.833, p = .002) and water access dry season (β = 0.782, p = .002).

Healthcare services exhibited a strong positive relationship with their indicators, as evidenced by high factor loadings for both delivered by (β = 0.901, p = .002) and delivered place (β = 0.865, p = .001). Regarding the structural pathways leading directly to child growth outcomes, minimum meal frequency (MMF) had a significant positive influence on acute growth (β = 0.107, p = .035), indicating that more frequent meals are associated with improved acute growth outcomes (higher WHZ scores). Crucially, the length of migration exerted a significant positive direct effect on acute growth (β = 0.155, p = .001), statistically validating that a shorter stay in border locations is directly associated with a higher risk of acute wasting (WHZ). Conversely, the paths from fever (β = -0.159, p = .003) and diarrhea (β = -0.125, p = .049) to acute growth (WHZ) were negative, suggesting that the presence of fever or diarrhea is linked to stunted acute growth (lower WHZ scores).

Other notable paths include HFI category and healthcare services both positively influencing minimum dietary diversity (MDD) (β = 0.190, p = .001 and β = 0.152, p = .015, respectively). Water availability has a strong positive effect on minimum meal frequency (MMF) (β = 0.294, p = .001), which implies that better water availability leads to more frequent meals. Furthermore, the length of migration also significantly predicted MMF (β = 0.127, p = .013), showing that longer-established residency enhances child meal frequency. Socioeconomic status (β = -0.219, p = .002) and HFI category (β = -0.125, p = .020) both have an inverse association with fever, suggesting that better socioeconomic conditions and food security are linked to a reduced incidence of fever. Socioeconomic status (β = -0.177, p = .002) and MDD (β = -0.125, p = .015) both have a negative association with diarrhea, indicating that better socioeconomic status and dietary diversity are associated with a lower incidence of diarrhea.

#### 3.2.3 Weight-for-age (WAZ) model.

The standardized path coefficients obtained from the weight-for-age (WAZ) model exhibited substantial congruence with the results from both the linear growth (HAZ) and acute growth (WHZ) models. Socioeconomic status was the strongest predictor of the household food insecurity (HFI) category (β = 0.318, p = .001). Furthermore, an increased number of children under five demonstrated an inverse relationship with the HFI category (β = -0.155, p = .002). Similarly, both Socioeconomic status (SES) (β = -0.180, p = .002) and minimum diet diversity (MDD) (β = -0.112, p = .034) exhibited a negative correlation with diarrhea, supporting the idea that higher SES and better MDD are protective against diarrhea. Furthermore, Socioeconomic status was negatively associated with acute respiratory infection (ARI) (β = -0.175, p = .001), indicating its broader influence on health outcomes.

Water availability was a powerful predictor of water access, with the strongest path to water access entire year (β = 0.959, p = .001). Socioeconomic status was highly correlated with both monthly income (β = 0.818, p = .001) and employment status (β = 0.790, p = .001). Healthcare services had strong positive paths to how and where care was delivered, with high coefficients for delivered by (β = 0.896, p = .001) and delivered place (β = 0.869, p = .002).

**Fig 2:**
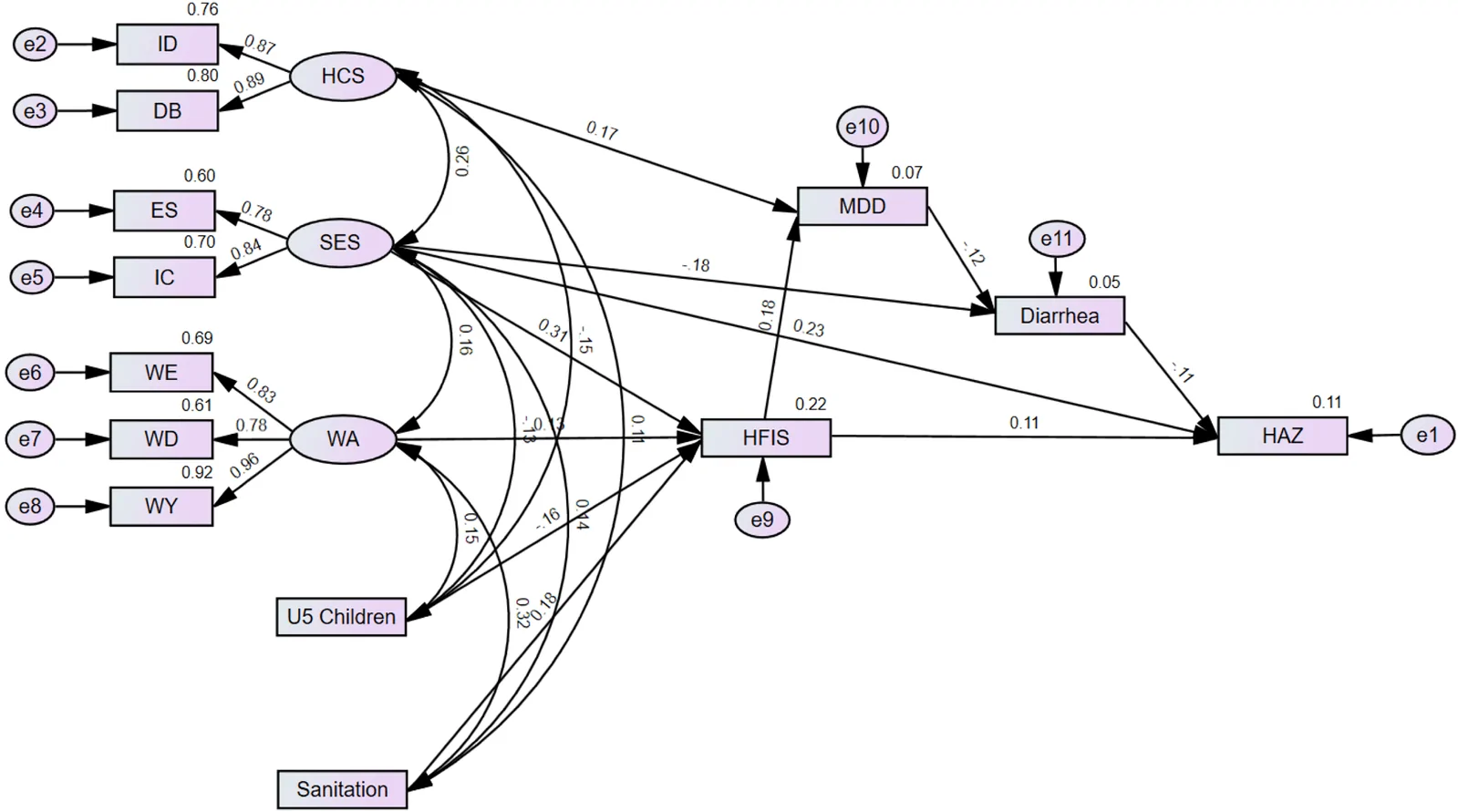
Standardized estimates on linear growth (HAZ) model. Structural Equation Models for linear growth (HAZ), acute growth (WHZ), and weight-for-age (WAZ). The diagrams illustrate the hypothesized pathways between socioeconomic, environmental, and health factors for each nutritional indicator. Circles (Ovals) represent latent variables (e.g., Socioeconomic Status, Healthcare Services, Water Availability), while rectangles indicate observed or measured variables (e.g., Monthly Income, U5 Children, Diarrhea, Fever). Single-headed arrows denote hypothesized direct causal paths, with standardized path coefficients (β) indicating the strength and direction of the relationship.

**Fig 3:**
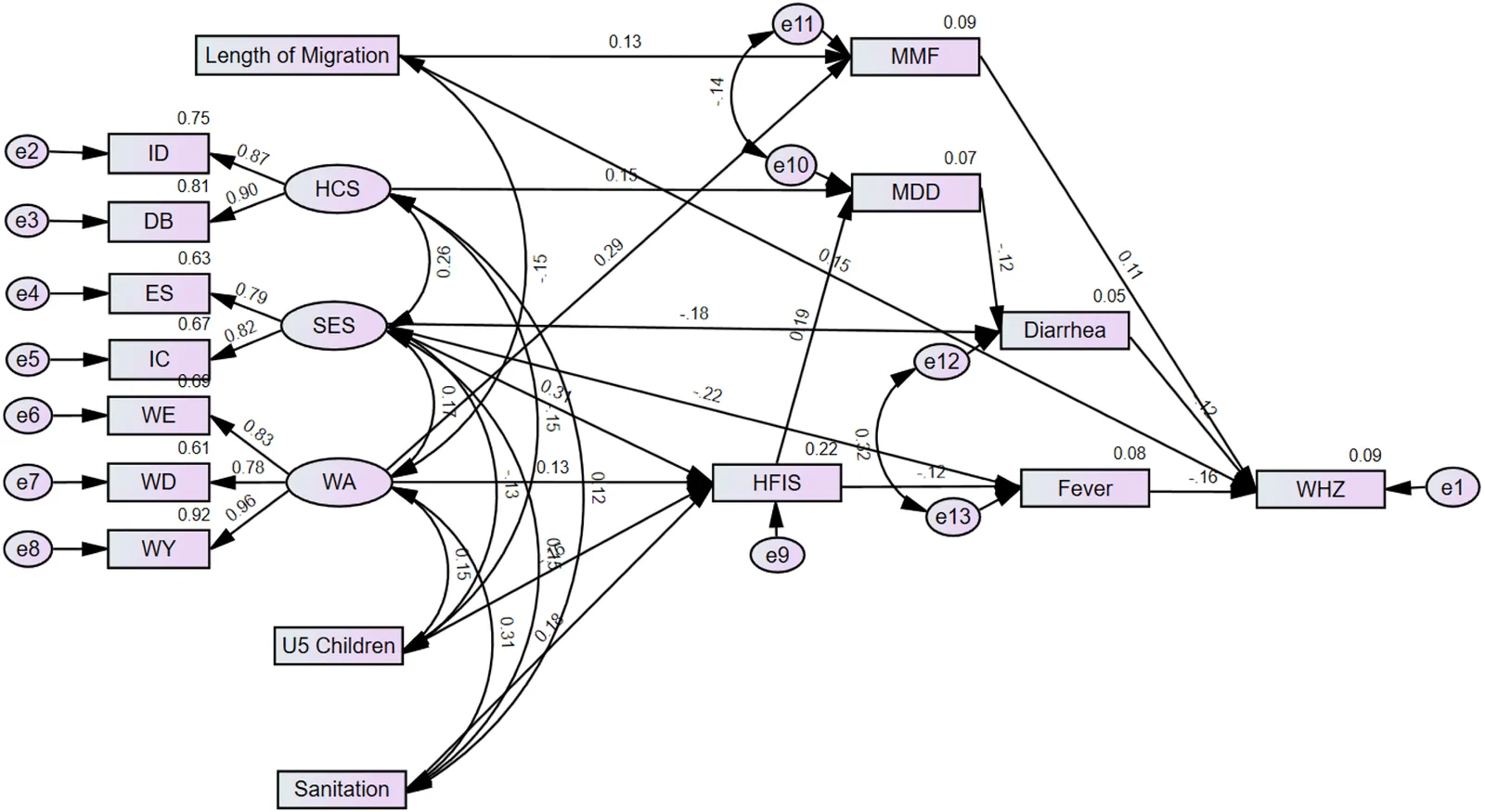
Standardized estimates on acute growth (WHZ) model. Structural Equation Models for linear growth (HAZ), acute growth (WHZ), and weight-for-age (WAZ). The diagrams illustrate the hypothesized pathways between socioeconomic, environmental, and health factors for each nutritional indicator. Circles (Ovals) represent latent variables (e.g., Socioeconomic Status, Healthcare Services, Water Availability), while rectangles indicate observed or measured variables (e.g., Monthly Income, U5 Children, Diarrhea, Fever). Single-headed arrows denote hypothesized direct causal paths, with standardized path coefficients (β) indicating the strength and direction of the relationship.

**Fig 4:**
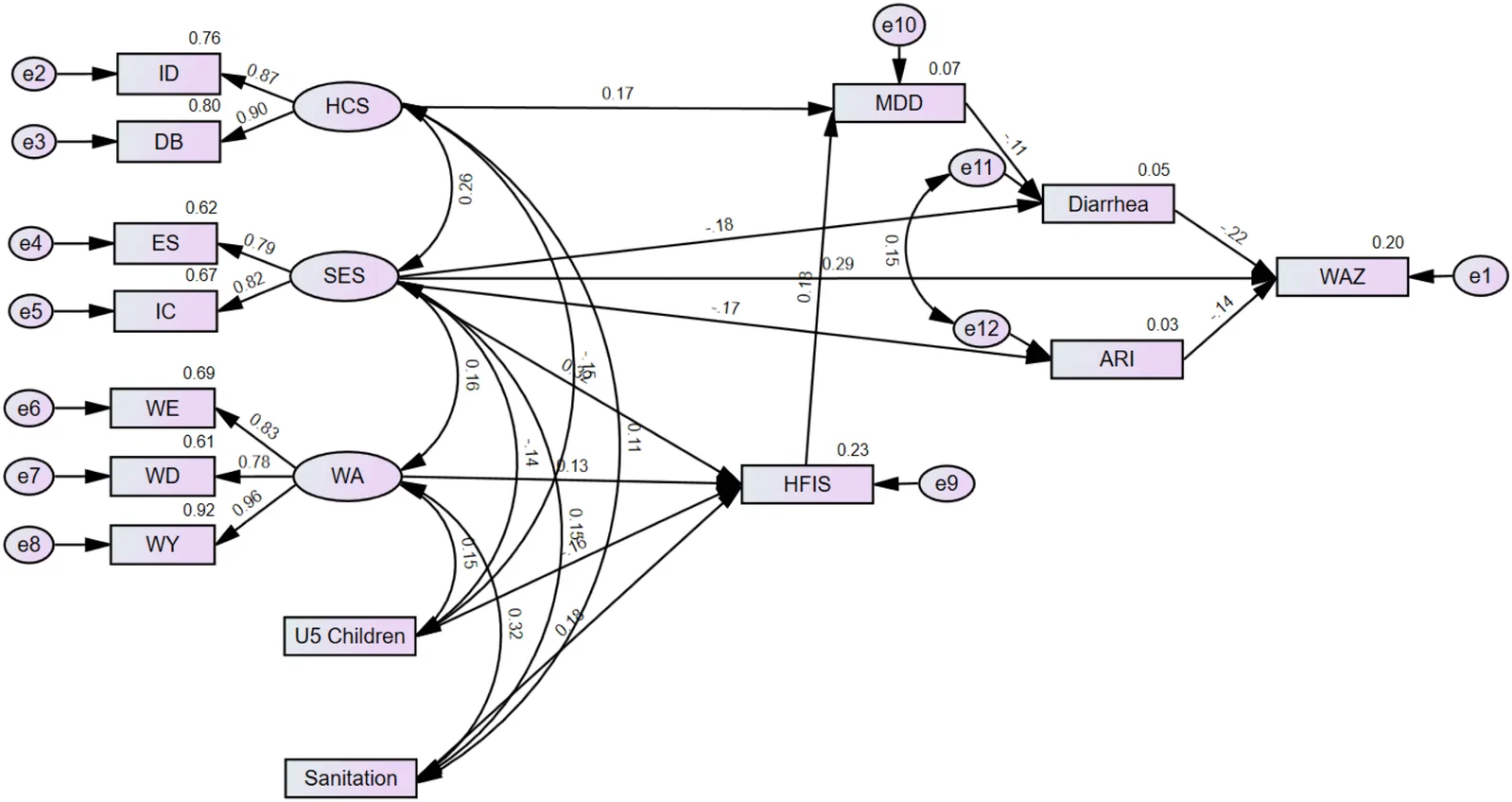
Standardized estimates on weight-for-age (WAZ) model. Structural Equation Models for linear growth (HAZ), acute growth (WHZ), and weight-for-age (WAZ). The diagrams illustrate the hypothesized pathways between socioeconomic, environmental, and health factors for each nutritional indicator. Circles (Ovals) represent latent variables (e.g., Socioeconomic Status, Healthcare Services, Water Availability), while rectangles indicate observed or measured variables (e.g., Monthly Income, U5 Children, Diarrhea, Fever). Single-headed arrows denote hypothesized direct causal paths, with standardized path coefficients (β) indicating the strength and direction of the relationship.

Socioeconomic status (SES) has a highly significant positive effect on weight-for-age (WAZ) (β = 0.294, p = .001). This indicates that children from higher socioeconomic backgrounds are more likely to have a higher WAZ scores, reflecting improved overall nutritional status. Conversely, the paths from diarrhea (β = -0.217, p = .001) and acute respiratory infection (ARI) (β = -0.144, p = .014) to weight-for-age (WAZ) were negative. These findings suggest that instances of diarrhea and respiratory illness are significantly associated with lower WAZ scores, thereby negatively impacting children's weight-for-age outcomes (Figs 2-Fig 3, Fig 4).

### 3.3 Mediation analysis, explanatory power, and model robustness

The structural equation model (SEM) results for direct, indirect, and total standardized effects are presented in **Table 5**. In the Linear Growth (HAZ) model, socioeconomic status (SES) emerged as the most dominant factor, exerting a total effect of 0.286 (p = .001). This was composed of a significant direct effect (β = 0.230, p = .001) and a significant indirect effect (β = 0.056, p = .004) mediated through household food insecurity and health factors. Household food insecurity also demonstrated a significant direct impact on HAZ (β = 0.113, p = .033). Among the distal factors, the number of children under five exerted a negative indirect effect (β = -0.018, p = .023), while safe sanitation (β = 0.021, p = .018) and water availability (β = 0.015, p = .023) contributed positively via indirect pathways. Regarding clinical factors, diarrhea was a significant direct predictor of lower HAZ (β = -0.109, p = .028). While healthcare services (β = 0.002, p = .023) and minimum dietary diversity (β = 0.013, p = .028) both exerted statistically significant but small total effects on HAZ.

**Table 5:** Direct, indirect, and total standardized effects on undernutrition (n = 384).

Path	Direct	Indirect	Total
**A: Linear Growth (HAZ) Model**			
Number of U5 Children -- > HAZ		-0.018 (.023)	-0.018 (.023)
Healthcare Services -- > HAZ		0.002 (.023)	0.002 (.023)
Safety Sanitation Practices -- > HAZ		0.021 (.018)	0.021 (.018)
Water Availability -- > HAZ		0.015 (.023)	0.015 (.023)
Socioeconomic Status -- > HAZ	0.230 (.001)	0.056 (.004)	0.286 (.001)
HFI Category -- > HAZ	0.113 (.033)	0.002 (.022)	0.116 (.030)
MDD -- > HAZ		0.013 (.028)	0.013 (.028)
Diarrhea -- > HAZ	-0.109 (.028)		-0.109 (.028)
**B: Acute Growth (WHZ) Model**			
Length of Migration -- > WHZ	0.155 (0.001)	0.013 (0.030)	0.168 (0.001)
Number of U5 Children -- > WHZ		-0.004 (0.003)	-0.004 (0.003)
Healthcare Services -- > WHZ		0.002 (0.023)	0.002 (0.023)
Safety Sanitation Practices -- > WHZ		0.004 (0.003)	0.004 (0.003)
Water Availability -- > WHZ		0.034 (0.020)	0.034 (0.020)
Socioeconomic Status -- > WHZ		0.064 (0.001)	0.064 (0.001)
HFI Category -- > WHZ		0.023 (0.004)	0.023 (0.004)
MDD -- > WHZ		0.016 (0.028)	0.016 (0.028)
MMF -- > WHZ	0.107 (0.035)		0.107 (0.035)
Fever -- > WHZ	-0.159 (0.003)		-0.159 (0.003)
Diarrhea -- > WHZ	-0.125 (0.049)		-0.125 (0.049)
**C: Weight-for-Age (WAZ) Model**			
Number of U5 Children -- > WAZ		-0.001 (.012)	-0.001 (.012)
Healthcare Services -- > WAZ		0.004 (.019)	0.004 (.019)
Safety Sanitation Practices -- > WAZ		0.001 (.014)	0.001 (.014)
Water Availability -- > WAZ		0.001 (.016)	0.001 (.016)
Socioeconomic Status -- > WAZ	0.294 (.001)	0.066 (.001)	0.360 (.001)
HFI Category -- > WAZ		0.004 (.018)	0.004 (.018)
MDD -- > WAZ		0.024 (.027)	0.024 (.027)
ARI -- > WAZ	-0.144 (.014)		-0.144 (.014)
Diarrhea -- > WAZ	-0.217 (.001)		-0.217 (.001)

The Acute Growth (WHZ) model emphasized immediate biological and dietary drivers. Notably, length of migration emerged as a primary determinant, exerting a significant positive direct effect (β = 0.155, p = .001) and a significant indirect effect (β = 0.013, p = .030), culminating in a strong positive total effect (β = 0.168, p = .001) on WHZ. This statistically reinforces that a shorter duration of migration strongly predicts a higher risk of acute growth (WHZ). For proximal health factors, recent morbidities were the strongest direct predictors of lower WHZ; fever demonstrated a significant negative direct and total effect (β = -0.159, p = .003), while diarrhea also maintained a negative direct and total path (β = -0.125, p = .049). Conversely, minimum meal frequency (MMF) played a critical protective role, showing a significant positive direct and total effect (β = 0.107, p = .035). Socioeconomic status influenced WHZ exclusively through indirect pathways (β = 0.064, p = .001). Environmental, dietary, and demographic factors also demonstrated significant indirect effects mediated through proximal behaviors and health statuses; these included water availability (β = 0.034, p = .020), household food insecurity category (β = 0.023, p = .004), safety sanitation practices (β = 0.004, p = .003), and minimum dietary diversity (β = 0.016, p = .028). Furthermore, healthcare services showed a small but significant positive, indirect and total effect on WHZ (β = 0.002, p = .023). In contrast, demographic pressure within the household, captured by the number of children under five, exerted a significant negative indirect and total effect on acute growth (β = -0.004, p = .003).

The Weight-for-Age (WAZ) model exhibited the highest cumulative influence from predictors. Socioeconomic status remained the dominant factor (β = 0.360, p = .001), driven by a strong direct path (β = 0.294, p = .001) and an indirect path (β = 0.066, p = .001). Clinical drivers were highly significant direct predictor (β = -0.217, p = .001) and acute respiratory infection (β = -0.144, p = .014). Minimum dietary diversity also demonstrated a significant direct effect (β = 0.024, p = .027). Similar to the other models, the number of children under five exerted a negative indirect effect (β = -0.001, p = .012). Other factors including household food insecurity category (β = 0.004), healthcare services (β = 0.004), sanitation (β = 0.001), and water availability (β = 0.001) contributed small though statistically significant total effects, indicating that their influence on WAZ is heavily mediated by health and dietary variables.

The R^2^ values, representing the proportion of variance explained for each endogenous variable, are presented in **Table 6**. The structural models accounted for 20.5% of the variance in WAZ, 11.0% in HAZ, and 9.3% in WHZ. Among the mediators, household food insecurity (HFI) demonstrated the highest explanatory power across all models (R^2^ ≈ .22), indicating that nearly a quarter of the variation in food insecurity is explained by the distal factors. In contrast, dietary mediators such as MDD (R^2^ ≈ .069) and MMF (R^2^ = .091), as well as morbidity factors like diarrhea (R^2^ ≈ .05), fever (R^2^ = .085), and ARI (R^2^ = .031), demonstrated lower variance explained. The measurement indicators for the latent constructs exhibited high reliability. Specifically, Year-round water access emerged as the strongest predictor (R^2^ ≈ .919), followed by delivered by (R^2^ ≈ .80) and delivered place (R^2^ ≈ .75). Socioeconomic indicators, including monthly income (R^2^ ≈ .70) and employment status (R^2^ ≈ .62), also showed substantial explanatory power within the measurement model.

**Table 6:** Coefficient of determination (R^2^) for the measurement and structural models.

Variable Type	Endogenous Variable	HAZ Model	WHZ Model	WAZ Model
Outcomes	Nutritional Status	.110	.093	.205
Mediators	Household Food Insecurity (HFI)	.222	.224	.226
	Minimum Dietary Diversity (MDD)	.069	.067	.069
	Minimum Meal Frequency (MMF)	–	.091	–
	Diarrhea	.051	.052	.049
	Fever	–	.085	–
	Acute Respiratory Infection (ARI)	–	–	.031
Indicators	Monthly Income	.701	.674	.669
	Employment Status	.601	.627	.625
	Water Access (Entire Year)	.919	.915	.919
	Water Enough (Past Month)	.694	.694	.693
	Water Access (Dry Season)	.601	.611	.625
	Delivered Place	.759	.748	.755
	Delivered By	.800	.811	.803

To ensure the stability of these findings, sensitivity analysis was conducted linear regression to examine the relationship between continuous HFIAS scores (range 0–27) and child nutritional outcomes **(Table E in** **S1 Appendix**). Higher HFIAS scores were significantly associated with lower HAZ (β = -0.090, SE = 0.029, p = .002) and WAZ (β = -0.042, SE = 0.019, p = .024). These results indicate that increased household food insecurity is linked to poorer long-term nutritional status among the sampled children. In contrast, the association between HFIAS scores and WHZ did not reach statistical significance (β = 0.016, p = .497). Overall, the direction and significance of these associations for HAZ and WAZ remain consistent with the primary SEM findings using categorical food insecurity variables, confirming the robustness of the study’s main results.

## 4 Discussions

This study illuminates the intricate pathways through which socioeconomic hardship, household food insecurity, and limited healthcare access cascade to drive child undernutrition within this vulnerable migrant community. The findings reveal a severe nutritional burden, highlighted by a 37.0% overall undernutrition prevalence among children aged 6–59 months. This substantial local burden is mirrored by large-scale empirical data from other low- and middle-income countries, where recent meta-analyses indicate that comparable indicators of growth failure affect approximately 26.42% of pediatric populations within similar resource-constrained contexts [32]. A particularly alarming finding is the high rate of acute growth failure (wasting) at 19.8%, which far surpasses the WHO South-East Asia regional average (14.7%) and Myanmar’s national average of approximately 7% [9,33]. This disparity suggests an acute nutritional crisis likely exacerbated by displacement and poor sanitation in the border zone. Conversely, the prevalence of linear growth failure (stunting) at 18.0% was lower than Myanmar's national average of 29%, while composite nutritional deficits (underweight) at 18.5% remained comparable to the national average of 19% [33]. Collectively, these results highlight the critical need for targeted nutritional interventions that address the unique structural vulnerabilities of migrant households.

Stunting was significantly associated with household economic stability and early-life care. Bivariate analysis revealed strong associations with employment status (p < .001) and monthly income (p < .001), with 91.3% of stunted children originating from households with unstable employment. These findings reflect how parental unemployment and financial hardship drive linear growth deficits [23,24,34,35]. When framed within the updated hierarchy of the UNICEF Conceptual Framework of the Determinants of Maternal and Child Nutrition (2020), these macro-level socioeconomic constraints act as structural enabling determinants that directly compromise household stability. Furthermore, household food insecurity (p < .001) was a strong predictor, with 39.1% of stunted children living in severely food-insecure households, corroborating research that household food insecurity significantly elevates the odds of chronic growth failure [36–38]. As a fundamental structural determinant, household food insecurity remains a persistent barrier to optimal biological and developmental potential throughout the life course, as documented across various life stages [39]. Under the UNICEF 2020 model, this food insecurity, alongside environmental conditions, operates at the household and community level as an underlying determinant of malnutrition.

Regarding birth and healthcare, stunting was linked to low birth weight (p = .028), delivery outside a health facility (p = .002), delivery by non-health professionals (p < .001), and incomplete routine immunization (p < .001). These findings align with evidence that low birth weight infants face more than double the risk of stunting compared to those with normal birth weights [35,40], while institutional delivery and complete vaccination coverage serve as critical protective factors [35,41–43]. The significant association with minimum dietary diversity (p = .002) confirms that dietary quality is fundamental in preventing chronic malnutrition [28,44,45]. Findings highlight a heavy reliance on starchy staples (92.7%) and a significant gap in the consumption of pulses, nuts, and seeds (58.9%). This disparity exposes a distinct dietary trade-off driven by economic constraints within unstable border labor environments. To protect their children from immediate hunger under severe financial stress, migrant households are structurally forced to prioritize immediate caloric satiety which filling hunger with inexpensive, accessible carbohydrates like white rice, over micronutrient density, which requires cost-prohibitive proteins, pulses, fruits, and vegetables. This behavioral shift directly operationalizes the consumer substitution principles under duress established in this study's theoretical foundation, demonstrating how severe resource scarcity compels a systematic substitution of dietary quality for energy adequacy as a primary survival mechanism [46,47]. Consequently, this economic coping behavior explains why household food insecurity acts as a persistent structural bottleneck that suppresses child dietary quality, locks vulnerable populations into a low-diversity diet, and ultimately impedes long-term linear growth.

The SEM of chronic growth failure revealed significant positive direct effects from socioeconomic status (β = 0.230, p = .001) and household food insecurity (β = 0.113, p = .033). These results underscore the vital importance of enduring economic well-being for linear growth [23,24,34–38]. The model reveals significant indirect pathways where socioeconomic status acts as a foundational determinant of child growth. Specifically, higher socioeconomic status improves linear growth outcomes (HAZ) by significantly reducing food insecurity (β = 0.311, p = .001), which serves as a key mediator for enhanced dietary diversity [44,48,49]. These results confirm that household food insecurity is a critical bridge between distal socioeconomic factors and proximal nutritional outcomes. Crucially, while the direct pathway from socioeconomic status to HAZ is already notable (β = 0.230, p = .001), its total effect is substantially compounded (β = 0.286, p = .001). This mathematical escalation validates the interlocking nature of the UNICEF framework, demonstrating how distal socioeconomic vulnerabilities sequentially accumulate and amplify as they cascade through household food access bottlenecks to drive absolute linear growth outcomes. The findings align with recent literature suggesting that the behavioral and diet-quality dimensions of household food insecurity are the primary pathways through which structural poverty translates into chronic growth failure [50]. Furthermore, the risk of household food insecurity in migrant contexts is strongly associated with socioeconomic characteristics, dietary diversity scores, and the volatility of food prices [51]. In addition to these economic drivers, environmental factors such as water availability (β = 0.131, p = .020) and sanitation practices (β = 0.181, p = .001) significantly impact the upstream mediator of household food insecurity, highlighting the cascading protective effect of a healthy environment on long-term nutrition [52–54].

Beyond the specific direct and indirect paths, latent construct correlations revealed critical background structural bottlenecks that remained highly consistent across the HAZ, WHZ, and WAZ models, reflecting systemic household vulnerabilities along the border. First, socioeconomic status exhibited a significant inverse relationship with the number of under-five children in the household (r = -.134 to -.140, p = .020). This negative covariance highlights a critical demographic trap where larger child dependency ratios directly deplete parental wealth resources, systematically locking larger migrant families into deeper financial strain. Second, higher socioeconomic status strongly covaried with better healthcare services access (r = .257 to .263, p < .001), while a higher density of young children was significantly linked to decreased healthcare utilization (r = -.149 to -.152, p = .006). These interrelated baseline configurations mathematically demonstrate that financial capital remains a strict prerequisite for navigating complex border healthcare systems, whereas the compounding daily burden of child-rearing creates severe physical and time mobility constraints that systematically restrict mothers from seeking timely care.

Wasting was most strongly associated with variables reflecting immediate economic volatility and morbidity. Higher wasting rates were significantly linked to employment status (p = .006), monthly income (p = .001), and mothers’ education (p = .046). This underscores that a lack of formal maternal education and parental unemployment leads to a substantial rise in the likelihood of acute malnutrition [23,34]. The most compelling driver of wasting was recent illness (p < .001); nearly 70% of wasted children had been ill in the two weeks preceding the survey. This confirms the direct, rapid impact of disease (e.g., fever or diarrhea) on acute weight loss [23,24,42].

Crucially, the length of migration emerged as a primary structural determinant of acute nutritional failure. The structural equation model validated that a shorter duration of migration is significantly linked to higher wasting risks, demonstrating a strong positive direct effect (β = 0.155, p = .001), a significant indirect effect (β = 0.013, p = .030), and a compounded positive total effect (β = 0.168, p = .001) on WHZ. This finding reflects the acute vulnerability associated with transient migration shocks and social capital displacement in border contexts [16,55]. Newly arrived migrants are heavily characterized by their unauthorized entry and lack of legal documentation due to conflict-driven displacement. Consequently, they experience a profound structural isolation, lacking fixed employer networks and possessing minimal institutional familiarity with Thai border healthcare services. This total detachment from both formal legal protections and informal community safety nets leaves infant-mother dyads uniquely exposed to sudden socioeconomic shocks and food access volatility. Statistically, the negative linear path confirms that the early phase of migration poses the highest operational and economic disruption, leaving newly arrived, unauthorized households exceptionally vulnerable to the rapid weight loss shocks that drive acute growth failure.

The structural model further validated the overarching conceptual framework by quantifying the simultaneous impacts of acute morbidity alongside these macro-migration factors. Both fever (β = -0.159, p = .003) and diarrhea (β = -0.125, p = .049) exerted significant direct effects, increasing a child's risk of a lower WHZ, a finding consistent with established literature [23,24,42]. In the UNICEF model, these acute morbidities and dietary limitations operate as immediate determinants at the individual child level. Rather than treating these pathways as purely clinical, they must be interpreted through the synergistic relationship between malnutrition and infection [3]. In temporary, under-resourced border settlements, sub-optimal water and sanitation infrastructure facilitate chronic subclinical pathogen ingestion. This acts as a primary catalyst for Environmental Enteric Dysfunction (EED), a condition that damages the intestinal lining through villous atrophy and triggers low-grade systemic inflammation [56–58]. Consequently, this subclinical malabsorption prevents the child's body from properly utilizing nutrients. Through this biological mechanism, the child suffers from acute nutritional deficits, leading to wasting (WHZ) even if a baseline level of food intake is successfully maintained.

Crucially, the model identifies two key protective pathways against acute growth failure. First, a vital nutritional pathway was revealed: better water availability significantly predicted higher minimum meal frequency (β = 0.294, p = .001), which in turn acted as a direct protective factor for WHZ (β = 0.107, p = .035). This highlights how an essential resource like water enables the consistent feeding practices necessary to prevent acute nutritional decline. Furthermore, the structural framework mapped a critical indirect cascade running from household food insecurity to acute growth failure via dietary quality and morbidity mediators. Within this multivariable system, minimum dietary diversity (MDD) does not dictate weight-for-height outcomes through an isolated direct path; instead, it functions as a pivotal behavioral buffer. Lower dietary diversity systematically deprives child systems of essential micronutrients, compromising gut mucosal integrity and systemic immunity. This nutritional deficit directly elevates susceptibility to infectious diarrhea, which subsequently acts as the proximal physiological catalyst driving rapid weight loss and acute growth failure. This statistical sequencing mathematically validates the classic malnutrition-infection cycle within marginalized border populations [14,16]. Intriguingly, latent construct evaluations within this WHZ framework uncovered a distinct negative association between minimum meal frequency (MMF) and minimum dietary diversity (MDD) (r = -.140, p = .007). This negative covariance captures a critical structural coping strategy and resource-constrained trade-off unique to vulnerable migrant households under severe economic duress. Specifically, financially constrained mothers often prioritize filling their child's stomach through daily meal frequency (maintaining MMF) using cheap, monotonous carbohydrate staples, at the direct expense of food variety and micronutrient density (sacrificing MDD) [48,59]. Additionally, a significant negative correlation emerged between water availability and the length of migration (r = -.151, p = .003), suggesting that newly arrived households face disproportionately worse baseline environmental barriers. Notably, socioeconomic status exhibited a significant inverse relationship with the disease mediators of diarrhea (β = -0.177, p = .002) and fever (β = -0.219, p = .002), reinforcing the protective role of economic stability against the infectious diseases that trigger a lower WHZ. Crucially, while the direct impact of socioeconomic status on acute growth failure (WHZ) is non-significant, its indirect and total effect coefficient is highly significant (β = 0.064, p = .001), mathematically demonstrating how distal socioeconomic disadvantages systematically accumulate as they cascade through proximal behaviors to dictate absolute acute nutritional outcomes [24,35,53].

Underweight status reflected a combination of chronic socioeconomic conditions and acute shocks. Significant associations were found with employment status (p < .001) and monthly income (p < .001), identifying these as primary drivers. These findings align with research linking household wealth and steady employment to child weight-for-age [60,61]. Additionally, household food insecurity was significantly linked to being underweight (p = .020), corroborating evidence that children in food-insecure households face higher odds of low weight-for-age [36–38]. Foundational early-life conditions including low birth weight (p = .003), preterm birth (p = .037), and being delivered by a non-health professional (p = .007), showed strong associations, with preterm birth particularly underscoring the lasting impact of gestational health [34,41]. Simultaneously, recent illness showed the strongest correlation with underweight (p < .001), affecting 76.1% of underweight children. This dual influence necessitates multi-sectoral interventions targeting both long-term developmental stability and rapid-response healthcare for acute morbidity [23].

The structural model for Weight-for-Age (WAZ) provides a comprehensive analysis of the factors influencing a child’s nutritional status, showing that socioeconomic status (β = 0.294, p = .001) exerts a strong positive direct effect. This aligns with global evidence identifying household poverty as a universal driver of lower WAZ across low- and middle-income countries [32,61]. Within this multivariable framework, socioeconomic status functions as a foundational determinant, impacting WAZ both directly and indirectly by reducing vulnerability to infectious disease mediators. When reviewing the total effects, the socioeconomic coefficient expands substantially to β = 0.360 (p = .001), demonstrating that macro-level inequalities act as the ultimate driver of composite biological deficits on the border. Additionally, acute illness mediators such as diarrhea (β = -0.217, p = .001) and acute respiratory infections (β = -0.144, p = .014) have a significant negative direct effect on WAZ. This dual pathway, which is well-established in research [23,60,62], highlights the complexity of addressing overall WAZ, requiring interventions that target both long-term developmental factors and short-term health crises. Furthermore, household food insecurity serves as a critical link in the model, confirming that economic hardship translates into nutritional decline through the food insecurity pathway (p = .020). In this migrant context, household food insecurity likely reflects income volatility, forcing a reliance on nutrient-poor staples that impede adequate WAZ improvement. These findings necessitate a multi-sectoral approach targeting both long-term food security and rapid-response healthcare to mitigate acute health crises [36–38].

The model demonstrated the highest explanatory power for composite nutritional deficits (underweight) (R^2^ = .205), confirming its utility as a sensitive indicator for the broad array of socioeconomic and morbidity factors analyzed. Interestingly, the variance explained for household food insecurity (R^2^ = .22) was higher than for specific biological outcomes, suggesting that the analyzed variables are stronger predictors of household food stability than of individual growth outcomes. This indicates that while the model successfully identifies households at risk of food access issues, individual biological health is likely influenced by a wider range of ecological variables beyond those captured in the current framework.

### 4.1 Strength and limitation of the study

The primary strength of this study is the application of Structural Equation Modeling (SEM), which permitted the simultaneous analysis of direct and mediated pathways within complex nutritional systems in a conflict-affected border zone. Furthermore, focusing on an under-researched migrant population provides context-specific data essential for targeted interventions, while the use of WHO Anthro Z-scores ensures international comparability.

Despite these strengths, several limitations are noted. The cross-sectional design precludes definitive causal inference; associations between food insecurity, morbidity, and growth may involve bidirectional relationships. For instance, while food insecurity affects feeding practices, chronic illness can also deplete household resources. Regarding external validity, the purposive selection of districts was necessitated by the sensitive border context and accessibility constraints that may limit generalizability, as the most remote or vulnerable households might be underrepresented. Additionally, the multi-stage sampling design may introduce community-level clustering that could influence the precision of standard errors. However, to ensure statistical precision and control for this community-level clustering effect during analysis, cluster-robust standard errors were utilized throughout the SEM executions, thereby mathematically adjusting the variance estimates across the surveyed settlement sites and ensuring the validity of the reported path significance (p-values). Statistically, the model showed modest explanatory power R^2^ for linear (R^2^ = .110) and acute growth failure (R^2^ = .093). These marginal values, combined with potential residual confounding from unmeasured variables such as maternal mental health or environmental enteric dysfunction [63], suggest that external factors significantly influence outcomes. Furthermore, acute wasting is often triggered by transient, seasonal shocks which a single temporal snapshot may fail to capture [32]. Future longitudinal studies are recommended to establish clearer temporal links and account for seasonal variability in migrant settings.

## 5 Conclusions

This study highlights a severe nutritional crisis among migrant children, with a wasting rate (19.8%) nearly triple Myanmar’s national average. Structural Equation Modeling (SEM) confirms that socioeconomic hardship translates into nutritional deficits primarily through household food insecurity; notably, nearly 40% of stunted children in this sample resided in severely food-insecure households. Crucially, the analysis identifies the length of migration as a pivotal structural determinant of acute nutritional deficits, demonstrating that a shorter duration of migration statistically compounds the risk of acute growth failure (WHZ). The model further demonstrates that while chronic growth failure (HAZ) is rooted in long-term food instability, acute growth failure (WHZ) and composite underweight status (WAZ) are heavily driven by the immediate physiological stress of morbidity. To address the central role of household food insecurity as a mediator of undernutrition, a multi-sectoral strategy is required. First, clinical interventions must prioritize the management of acute illnesses, specifically diarrhea and fever. Second, social protection mechanisms must prioritize newly arrived migrant families who bear the brunt of recent migration shocks; targeted programs, including emergency cash transfers, legal documentation assistance, and stable employment initiatives, are essential to mitigate the extreme nutritional vulnerability caused by initial displacement and income volatility. Third, nutritional programs should promote affordable, shelf-stable protein sources, such as pulses, to bridge dietary diversity gaps. Fourth, strengthening WASH infrastructure remains critical for both disease prevention and improved dietary quality. These interventions must be integrated across healthcare, social protection, and environmental health sectors to ensure a synergistic impact. Finally, further research utilizing longitudinal designs and biomarkers is required to explore sub-clinical pathways, such as gut health, to fully elucidate the etiology of growth failure in migrant populations.

## Supporting information

S1 Appendix Table A. Measurement Model Evaluation. **Table B.** Discriminant Validity of Latent Constructs (Fornell-Larcker Criterion). **Table C.** Correlations and Significance Levels for all Model Variables. **Table D.** Distribution of Dietary Consumption. **Table E.** Sensitivity Analysis (Continuous HFIAS Regression Results).
(DOCX)

S1 Data Minimal underlying dataset. (XLSX)

## References

[1] FAO, IFAD, UNICEF, WFP, WHO. The State of Food Security and Nutrition in the World 2024 – Financing to End Hunger, Food Insecurity and Malnutrition in All Its Forms. Rome: Food and Agriculture Organization of the United Nations. 2024. doi: 10.4060/cd1254en

[2] World Health Organization. Malnutrition. Geneva: WHO. 2024.

[3] UNICEF. Nutrition, for every child: UNICEF nutrition strategy 2020–2030. New York: UNICEF. 2020.

[4] Black MM, Lutter CK, Trude ACB. All children surviving and thriving: re-envisioning UNICEF’s conceptual framework of malnutrition. Lancet Glob Health. 2020;8(6):e766–7. doi: 10.1016/S2214-109X(20)30122-4 32446344

[5] World Bank Group. World Bank Support to Reducing Child Undernutrition. Washington (DC): World Bank. 2023.

[6] UNICEF. Conceptual Framework on the Determinants of Maternal and Child Nutrition: A Framework for the Prevention of Malnutrition in All Its Forms. New York: UNICEF. 2021.

[7] Zembe-Mkabile W. Social protection as a nutrition-sensitive instrument to address malnutrition in sub-Saharan Africa: Examining the utility of the UNICEF conceptual model of care for maternal and child nutrition. J int comp soc policy. 2023;39(3):295–305. doi: 10.1017/ics.2024.5

[8] UNICEF, World Health Organization, World Bank Group. Joint child malnutrition estimates, 2023 edition. New York: UNICEF. 2023.

[9] World Health Organization. World health statistics 2024: Monitoring health for the SDGs, Sustainable Development Goals. Geneva: WHO. 2024.

[10] UNICEF. Health and Nutrition: Working to Improve Access to Child and Maternal Health and Nutrition. New York: UNICEF. 2024.

[11] Maffioli EM, Headey D, Lambrecht I, Oo TZ, Zaw NT. A prepandemic nutrition-sensitive social protection program has sustained benefits for food security and diet diversity in Myanmar during a severe economic crisis. The Journal of Nutrition. 2023;153(4):1052–62. doi: 10.1016/j.tjnut.2023.02.009 36792031

[12] UNICEF. Country profiles: Myanmar. UNICEF. 2024.

[13] UNICEF. Myanmar humanitarian appeal for children 2026. Yangon: UNICEF Myanmar Country Office. 2026.

[14] Hashmi AH, Nyein PB, Pilaseng K, Paw MK, Darakamon MC, Min AM, et al. Feeding practices and risk factors for chronic infant undernutrition among refugees and migrants along the Thailand-Myanmar border: a mixed-methods study. BMC Public Health. 2019;19(1):1586. doi: 10.1186/s12889-019-7825-7 31779599 PMC6883662

[15] The Border Consortium. Nutrition survey of refugee camps along the Thailand–Myanmar border. TBC. 2022.

[16] Praditsor P, Churak P, Wimonpeerapattana W, Moore T, Bovill M. Prevalence of undernutrition and associated factors among children 6 to 59 months of age in refugee camps along Thailand-Myanmar border. Mahidol University. 2019.

[17] World Health Organization. WHO child growth standards: methods and development. Geneva: WHO. 2016.

[18] World Health Organization. Nutrition landscape information system (NLiS), country profile indicators interpretation guide. Geneva: WHO. 2019.

[19] World Health Organization. WHO Anthro for personal computers, version 3.2.2: Software for assessing growth and development of the world’s children. Geneva: WHO. 2011.

[20] de Onis M, Borghi E, Arimond M, Webb P, Croft T, Saha K, et al. Prevalence thresholds for wasting, overweight and stunting in children under 5 years. Public Health Nutr. 2019;22(1):175–9. doi: 10.1017/S1368980018002434 30296964 PMC6390397

[21] Seyoum K, Tekalegn Y, Quisido B. Determinants and prevalence of early initiation of breastfeeding: Does the place of delivery matter? A comparative cross-sectional study based on the 2016 Ethiopian demographic and health survey data. Popul Med. 2021;3(December):1–8. doi: 10.18332/popmed/144318

[22] Bilinsky JCASP. Household Food Insecurity Access Scale (HFIAS) for Measurement of Food Access: Indicator Guide. Washington (DC): Food and Nutrition Technical Assistance Project, Academy for Educational Development. 2007.

[23] Rahman MM, Kiyu A, Seling NR. Prevalence and factors associated with undernutrition among Dayak children in rural areas of Sarawak, Malaysia. Malaysian Journal of Nutrition. 2021;27(3):433–48. doi: 10.31246/mjn-2021-0045

[24] Rahimi BA, Khalid AA, Lali WM, Khalid WA, Rahimi JA, Taylor WR. Prevalence and associated risk factors of stunting, wasting/thinness, and underweight among primary school children in Kandahar City, Afghanistan: a cross-sectional analytical study. BMC Public Health. 2024;24(1):2321. doi: 10.1186/s12889-024-19858-z 39192206 PMC11348593

[25] World Health Organization. Indicators for assessing infant and young child feeding practices: part 2 measurement. Geneva: World Health Organization. 2010.

[26] Tebeje TM, Abebe M, Tesfaye SH, Seboka BT, Argaw GS, Seifu BL, et al. Minimum meal frequency and associated factors among children aged 6-23 months in Sub-Saharan Africa: a multilevel analysis of the demographic and health survey data. Front Public Health. 2024;12:1468701. doi: 10.3389/fpubh.2024.1468701 39651478 PMC11621055

[27] FAO. Guidelines for measuring household and individual dietary diversity. Roma: FAO. 2013.

[28] Saha J, Chouhan P, Malik NI, Ghosh T, Das P, Shahid M, et al. Effects of Dietary Diversity on Growth Outcomes of Children Aged 6 to 23 Months in India: Evidence from National Family and Health Survey. Nutrients. 2022;15(1):159. doi: 10.3390/nu15010159 36615816 PMC9824371

[29] FAO. Global strategic framework for food security and nutrition. 2011.

[30] Jöreskog K, Olsson U, Wallentin F, St C. Multivariate analysis with LISREL. Cham: Springer Nature. 2022.

[31] Hu L, Bentler PM. Cutoff criteria for fit indexes in covariance structure analysis: Conventional criteria versus new alternatives. Structural Equation Modeling: A Multidisciplinary Journal. 1999;6(1):1–55. doi: 10.1080/10705519909540118

[32] Dassie GA, Chala Fantaye T, Charkos TG, Sento Erba M, Balcha Tolosa F. Factors influencing concurrent wasting, stunting, and underweight among children under five who suffered from severe acute malnutrition in low- and middle-income countries: a systematic review. Front Nutr. 2024;11:1452963. doi: 10.3389/fnut.2024.1452963 39713780 PMC11660920

[33] Ministry of Health and Sports (MOHS), ICF International. Myanmar demographic and health survey 2015-16. Nay Pyi Taw (Myanmar): Ministry of Health and Sports (MOHS). 2016.

[34] Khan S, Zaheer S, Safdar NF. Determinants of stunting, underweight and wasting among children < 5 years of age: evidence from 2012-2013 Pakistan demographic and health survey. BMC Public Health. 2019;19(1):358. doi: 10.1186/s12889-019-6688-2 30935382 PMC6444880

[35] Blankenship JL, Cashin J, Nguyen TT, Ip H. Childhood stunting and wasting in Myanmar: Key drivers and implications for policies and programmes. Matern Child Nutr. 2020;16 Suppl 2(Suppl 2):e12710. doi: 10.1111/mcn.12710 32835450 PMC7591306

[36] Berra WG. Household Food Insecurity Predicts Childhood Undernutrition: A Cross-Sectional Study in West Oromia (Ethiopia). J Environ Public Health. 2020;2020:5871980. doi: 10.1155/2020/5871980 32211049 PMC7085371

[37] Rahman MM, Seling NR, Kiyu A. Under-five nutritional status and its relationship with household dietary diversity and food security among the Dayak communities in Sarawak, Malaysia. Bangladesh Medical Research Council Bulletin. 2022. doi: 10.3329/bmrcb.v47i2.57770

[38] Ali D, Saha KK, Nguyen PH, Diressie MT, Ruel MT, Menon P, et al. Household food insecurity is associated with higher child undernutrition in Bangladesh, Ethiopia, and Vietnam, but the effect is not mediated by child dietary diversity. J Nutr. 2013;143(12):2015–21. doi: 10.3945/jn.113.175182 24089419

[39] Tari Selcuk K, Atan RM, Arslan S, Sahin N. Relationship between food insecurity and geriatric syndromes in older adults: A multicenter study in Turkey. Exp Gerontol. 2023;172:112054. doi: 10.1016/j.exger.2022.112054 36513213

[40] Guirindola MO, Goyena EA, Maniego MLV. Risk factors of stunting during the complementary feeding period 6–23 months in the Philippines. Mal J Nutr. 2021;27(1):123–40. doi: 10.31246/mjn-2020-0112

[41] Kang Y, Kim J. Risk factors for undernutrition among children 0-59 months of age in Myanmar. Matern Child Nutr. 2019;15(4):e12821. doi: 10.1111/mcn.12821 PMC6859997 30919554

[42] Sartika AN, Khoirunnisa M, Meiyetriani E, Ermayani E, Pramesthi IL, Nur Ananda AJ. Prenatal and postnatal determinants of stunting at age 0–11 months: A cross-sectional study in Indonesia. PLOS ONE. 2021;16(7):e0254662. doi: 10.1371/journal.pone.0254662 PMC8279365 34260622

[43] Dadras O, Suwanbamrung C, Jafari M, Stanikzai MH. Prevalence of stunting and its correlates among children under 5 in Afghanistan: the potential impact of basic and full vaccination. BMC Pediatr. 2024;24(1):436. doi: 10.1186/s12887-024-04913-w 38971723 PMC11227132

[44] Aboagye RG, Seidu AA, Ahinkorah BO, Arthur-Holmes F, Cadri A, Dadzie LK. Dietary diversity and undernutrition in children aged 6-23 months in sub-Saharan Africa. Nutrients. 2021;13(10):3431. doi: 10.3390/nu13103431 34684435 PMC8537414

[45] Paramashanti BA, Paratmanitya Y, Kusumaningtyas II, Khasana TM, Yugistyowati A, Siswati T. Minimum dietary diversity and the concurrence of stunting and overweight among infants and young children in Yogyakarta, Indonesia. NFS. 2023;54(1):120–30. doi: 10.1108/nfs-02-2023-0042

[46] Yang Y-W, Liu J, Wang Y. Consumers’ variety-seeking behaviors under time pressure: Based on regulatory focus and excitement levels. Psych J. 2024;13(3):440–55. doi: 10.1002/pchj.770 38747182 PMC11169758

[47] Mennekes T, Schramm-Klein H. Effects of crisis-induced inflation on purchasing and consumer behavior in Germany. Journal of Retailing and Consumer Services. 2025;85:104295. doi: 10.1016/j.jretconser.2025.104295

[48] Yadav SS, Matela H, Panchal P, Menon K. Household food insecurity, dietary diversity with undernutrition among children younger than five years in Indian subcontinent-a narrative review. Lancet Reg Health Southeast Asia. 2024;26:100426. doi: 10.1016/j.lansea.2024.100426 38946926 PMC11214174

[49] Khura B, Ahmed KY, Mohanty P, Kumar CP, Thapa S. Minimum dietary diversity is associated with lower risk of childhood underweight: Evidence from the 2019/2021 National Family Health Survey of India. Nutr Res. 2024;130:11–21. doi: 10.1016/j.nutres.2024.08.003 39303360

[50] Tari Selcuk K, Atan RM, Arslan S, Sahin N. Is food insecurity related to sustainable and healthy eating behaviors?. Environ Sci Pollut Res Int. 2023;30(29):74280–9. doi: 10.1007/s11356-023-27694-8 37204579 PMC10197049

[51] Shuvo SD, Hossain MS, Riazuddin M, Mazumdar S, Roy D. Factors influencing low-income households’ food insecurity in Bangladesh during the COVID-19 lockdown. PLoS One. 2022;17(5):e0267488. doi: 10.1371/journal.pone.0267488 35536850 PMC9089875

[52] Novianti S, Huriyati E, Padmawati RS. Safe Drinking Water, Sanitation and Mother’s Hygiene Practice as Stunting Risk Factors: A Case Control Study in a Rural Area of Ciawi Sub-district, Tasikmalaya District, West Java, Indonesia. Ethiop J Health Sci. 2023;33(6):935–44. doi: 10.4314/ejhs.v33i6.3 38784492 PMC11111280

[53] Rahut DB, Mishra R, Bera S. Geospatial and environmental determinants of stunting, wasting, and underweight: Empirical evidence from rural South and Southeast Asia. Nutrition. 2024;120:112346. doi: 10.1016/j.nut.2023.112346 38320385

[54] Soe TK, Laohasiriwong W, Sornlorm K, Mahato RK. Safely managed sanitation practice and childhood stunting among under five years old children in Myanmar. PLoS One. 2023;18(11):e0290600. doi: 10.1371/journal.pone.0290600 37983207 PMC10659194

[55] Roshania RP, Giri R, Cunningham SA, Young MF, Webb-Girard A, Das A, et al. Early life migration and undernutrition among circular migrant children: An observational study in the brick kilns of Bihar, India. J Glob Health. 2022;12:04008. doi: 10.7189/jogh.12.04008 35136599 PMC8818295

[56] Victora CG, Christian P, Vidaletti LP, Gatica-Domínguez G, Menon P, Black RE. Revisiting maternal and child undernutrition in low-income and middle-income countries: variable progress towards an unfinished agenda. Lancet. 2021;397(10282):1388–99. doi: 10.1016/S0140-6736(21)00394-9 33691094 PMC7613170

[57] Carvalho MCC, Ribeiro SA, de Sousa LS, Lima AÂM, Maciel BLL. Undernutrition and Intestinal Infections in Children: A Narrative Review. Nutrients. 2025;17(9):1479. doi: 10.3390/nu17091479 40362788 PMC12073655

[58] Htet MK, Nurfadilah M, Angelin TC, Momo Kadia B, Gabain IL, Ramsteijn AS, et al. Environmental enteric dysfunction influences linear growth of children through insulin-like growth factor-1: findings from the Indonesian Action Against Stunting Hub birth cohort. Philos Trans R Soc Lond B Biol Sci. 2026;381(1950):20250041. doi: 10.1098/rstb.2025.0041 42132033

[59] Tauseef SL, Khin Mar, Oo T, van Asselt J. The state of food security and nutrition in Myanmar, 2021-2025: Findings from nine rounds of the Myanmar Household Welfare Survey. International Food Policy Research Institute. 2026.

[60] Manoochehri S, Faradmal J, Poorolajal J, Asadi FT, Soltanian AR. Risk factors associated with underweight in children aged one to two years: a longitudinal study. BMC Public Health. 2024;24(1):1875. doi: 10.1186/s12889-024-19147-9 39004703 PMC11247798

[61] Birhanu F, Yitbarek K, Bobo FT, Atlantis E, Woldie M. Undernutrition in children under five associated with wealth-related inequality in 24 low- and middle-income countries from 2017 to 2022. Sci Rep. 2024;14(1):3326. doi: 10.1038/s41598-024-53280-0 38336795 PMC10858243

[62] Tamir TT, Zegeye AF, Workneh BS, Mekonen EG. Underweight and associated factors among children under age of five in low and lower-middle income African countries: hierarchical analysis of demographic and health survey data. Front Public Health. 2024;12:1423603. doi: 10.3389/fpubh.2024.1423603 39314788 PMC11417020

[63] Lowe C, Tsheten T, Wagnew F, Sarma H, Ancha A, Gray D, et al. Biomarkers of environmental enteric dysfunction associated with the linear growth of children 0-5 years in low- and middle-income countries: a systematic review. Nutr Res Rev. 2025;39:e3. doi: 10.1017/S0954422425100231 41177769

