# Supplementary material for: Pathways linking household food insecurity to undernutrition among under 5 children in migrant populations along the Thailand-Myanmar border: A Structural Equation Modeling approach
Source: PLOS Glob Public Health. 2026 Aug 20;6(8):e0006516. doi: 10.1371/journal.pgph.0006516 (PMC13492760; doi:10.1371/journal.pgph.0006516)
Supplement: Supplementary file 1 [file pgph.0006516.s001.docx]

**Supporting Information: S1 Appendix**

**Title:** Pathways Linking Household Food Insecurity to Undernutrition Among Under 5 Children in Migrant Populations Along the Thailand-Myanmar Border: A Structural Equation Modeling Approach

**Author:** Saw Min Than

**Corresponding Author:** Cheerawit Rattanapan, Email: [cheerawit.rat@mahidol.ac.th](mailto:cheerawit.rat@mahidol.ac.th)

**Contents:**

1. **Table A.** Measurement Model Evaluation.
2. **Table B.** Discriminant Validity of Latent Constructs (Fornell-Larcker Criterion).
3. **Table C.** Correlations and Significance Levels for all Model Variables.
4. **Table D.** Distribution of Dietary Consumption.
5. **Table E.** Sensitivity Analysis (Continuous HFIAS Regression Results).

**Table A.** Measurement Model Evaluation.

*This table supports Measurement Model: Validity and Correlation Analysis in Section 2.7.2.*

| **Latent Constructs and Indicators** | **Unstandardized (B)** | **SE** | **Standardized (β)** | **P-value** | **AVE** | **CR** |
| --- | --- | --- | --- | --- | --- | --- |
| Socioeconomic status (SES) |  |  |  |  | 0.650 | 0.943 |
| Employment status (ES) | 1.000^a^ |  | 0.797 | <.001*** |  |  |
| Monthly income (IC) | 0.994 | 0.189 | 0.815 | <.001*** |  |  |
| Healthcare services (HCS) |  |  |  |  | 0.782 | 0.989 |
| Delivered by (DB) | 1.000^a^ |  | 0.922 | <.001*** |  |  |
| Delivered place (Institutional) (ID) | 1.050 | 0.183 | 0.845 | <.001*** |  |  |
| Water Availability (WA) |  |  |  |  | 0.740 | 0.906 |
| Water enough past month (WE) | 1.000^a^ |  | 0.825 | <.001*** |  |  |
| Water access dry season (WD) | 1.006 | 0.057 | 0.775 | <.001*** |  |  |
| Water access entire year (WY) | 1.166 | 0.056 | 0.969 | <.001*** |  |  |

***Note:*** *B = Unstandardized loading; SE = Standard Error; β = Standardized loading; AVE = Average Variance Extracted; CR = Composite Reliability. ^a^; Unit variance was constrained by fixing the first indicator of each construct to 1.0 (Marker Indicator). *** p < 0.001.* *Recommended thresholds: Standardized loadings (β) > 0.70, AVE* **≥** *0.50, and CR* **≥** *0.70.*

**Table B.** Discriminant Validity of Latent Constructs (Fornell-Larcker Criterion).

*This table supports Measurement Model: Validity and Correlation Analysis in Section 2.7.2.*

| **Latent Constructs** | **1. SES** | **2. HCS** | **3. WA** |
| --- | --- | --- | --- |
| 1. Socioeconomic status (SES) | (0.806) | - | - |
| 2. Healthcare services (HCS) | 0.248 | (0.884) | - |
| 3. Water Availability (WA) | 0.155 | 0.000 | (0.860) |

***Note:*** *Note: Diagonal values (in parentheses) are the square root of the AVE; off-diagonal values are the inter-construct correlations.*

**Table C.** Correlations and Significance Levels for all Model Variables.

*This table supports Measurement Model: Validity and Correlation Analysis in Section 2.7.2.*

| **Covariance** | **HAZ (r, p)** | **WHZ (r, p)** | **WAZ (r, p)** |
| --- | --- | --- | --- |
| Socioeconomic Status <--> Water Availability | .157** (.008) | .168** (.004) | .164** (.006) |
| Socioeconomic Status <--> Safety Sanitation Practices | .141* (.014) | .146* (.011) | .146* (.011) |
| Socioeconomic Status <--> Healthcare Services | .257*** (<.001) | .258*** (<.001) | .263*** (<.001) |
| Socioeconomic Status <--> Number of U5 Children | -.134* (.019) | -.134* (.020) | -.140* (.015) |
| Water Availability <--> Number of U5 Children | .148** (.004) | .148** (.003) | .148** (.004) |
| Water Availability <--> Safety Sanitation Practices | .318*** (<.001) | .308*** (<.001) | .318*** (<.001) |
| Water Availability <--> Length of Migration | - | -.151** (.003) | - |
| Safety Sanitation Practices <--> Healthcare Services | .114* (.028) | .118* (.023) | .114* (.028) |
| Healthcare Services <--> Number of U5 Children | -.152** (.006) | -.149** (.006) | -.151** (.006) |
| Diarrhea <--> Fever | - | .315*** (<.001) | - |
| Diarrhea <--> ARI | - | - | .148** (.005) |
| MMF <--> MDD | - | -.140** (.007) | - |

*Note: r; Standardized correlation coefficient, p; Significant value (Significant at * p < .05, ** p < .01, *** p < .001)*

**Table D.** Distribution of Dietary Consumption.

*This table supports Distribution of Dietary Consumption in Section 3.1 (Bivariate Analysis).*

| **Food Group** | **Frequency (%)** |
| --- | --- |
| Grains, roots, tubers and plantains | 356 (92.7) |
| Pulses (beans, peas, lentils), nuts, and seeds | 226 (58.9) |
| Diary products (milk, infant formula, yogurt, cheese) | 266 (69.3) |
| Flesh foods (meat, fish, poultry, organ meats) | 316 (82.3) |
| Eggs | 290 (75.5) |
| Vitamin-A rich fruits and vegetables | 253 (65.9) |
| Other fruits and vegetables | 263 (68.5) |

**Table E.** Sensitivity Analysis (Continuous HFIAS Regression Results).

*This table supports the model robustness check in Section 3.3.*

| **Outcome Variable** | **β Coefficient (HFIAS Score)** | **Standard Error** | **p-value** |
| --- | --- | --- | --- |
| Height-for-age z-score (HAZ) | -0.090 | .029 | .002 |
| Weight-for-height z-score (WHZ) | 0.016 | .024 | .497 |
| Weight-for-age z-score (WAZ) | -0.042 | .019 | .024 |

*Note: Linear regression models were used with continuous HFIAS score (range 0–27). Negative coefficients indicate that higher household food insecurity is associated with poorer child nutritional outcomes.*
